# Genome-Wide Identification of *NLR* Gene Family and Expression Patterns in Response to *Colletotrichum camelliae* Infection in Tea Plant (*Camellia sinensis*)

**DOI:** 10.3390/genes17101302

**Published:** 2026-10-20

**Authors:** Jin Wang, Annan Li, Qianzhu Wan, Yifei Zhang, Jun Tang, Qinxue Zhang

**Affiliations:** 1 School of Biological Science and Technology, Jiangsu University, Zhenjiang 212013, China; 2 Jiangsu Key Laboratory for Horticultural Crop Genetic Improvement, Institute of Leisure Agriculture, Jiangsu Academy of Agricultural Sciences, Nanjing 210014, China

**Keywords:** tea plant, *Colletotrichum camelliae*, *NLR* gene family, bioinformatics analysis, expression patterns

## Abstract

**Background**: *Colletotrichum camelliae* is an important fungal disease in tea plants (*Camellia sinensis*), and *NLR* gene family play a central role in plant disease resistance recognition. Analyzing the expression characteristics and regulatory network of *NLR* genes in tea plants in response to *anthracnose* infection can provide a theoretical basis for exploring key genes for tea plant resistance to *anthracnose* and elucidating the molecular mechanisms of disease resistance. **Method**: Using the tea tree reference genome Shuchazao 2 as the material, *CsNLR* genes were identified based on NB-ARC (Pfam ID: PF00931) and LRR (Pfam ID: PF00560) conserved domains; Conduct bioinformatics analysis using TBtools, MEME, and MEGA; Using quantitative real-time PCR (qRT-PCR) to detect the expression changes of CsNLR in the common variety Sucha 120 and the purple variety Ziying 2 after *anthracnose* infection, and constructing a protein prediction association network. **Result**: A total of 158 *CsNLR* genes were identified, and their physicochemical properties, phylogenetics, gene structure, and cis element analysis revealed differentiation among family members. The promoters generally contain stress and hormone response elements; CsNLR is highly expressed in roots, stems, and mature leaves. After 4 days of inoculation with *anthracnose* infection, the expression trends of *CsNLR* in the two tea tree varieties were opposite, with Sucha 120 upregulated and Ziying 2 downregulated; The predicted protein association network shows *CsNLR* as the central node of the network, with 77 CsNLR proteins were included in the predictive functional association network, and there were differences in the connectivity of different members. **Conclusions**: The *CsNLR* identified in this study is a potential *NLR* resistant gene for *anthracnose* in tea plants, laying the foundation for subsequent functional verification of disease resistant genes and analysis of resistance mechanisms.

## 1. Introduction

The tea plant (*Camellia sinensis*) belongs to the *Camellia genus* of the Theaceae family and is a perennial evergreen woody plant. It is also an important economic forest in China and is widely introduced and cultivated worldwide [1]. Tea, as the second most consumed beverage in the world after water, is highly favored for its unique flavor and various health benefits. This characteristic is mainly derived from the bioactive components such as caffeine [2], catechins [3], and theanine [4] contained in tea. These substances not only constitute the taste and aroma of tea, but also endow it with multiple physiological functions such as refreshing, antioxidant, and immune regulation [5]. Tea plant cultivation has significant economic value. As the world’s largest producer and consumer of tea, China’s tea industry has become a pillar industry of agricultural economy in many regions [6,7]. During the cultivation process of tea plant, they are often affected by various biological stresses. Tea *anthracnose*, caused by *Colletotrichum* spp., is among the most destructive foliar fungal diseases in Chinese tea plantations, inflicting 30–50% yield losses annually. *Anthracnose* is a global plant fungal disease caused by semi living nutritional fungi of the *Colletotrichum genus* in the *Ascomycota phylum*. *Anthracnose* has a wide host range and can infect fruit trees, vegetables, oilseeds, and woody economic crops. It is highly prone to outbreak under warm and high humidity conditions, causing leaf necrosis and fruit decay, seriously reducing agricultural product yield and quality [8]. The fungal pathogens mainly infect the mature and old leaves of tea plant, with typical symptoms of irregular brown to grayish white disease spots on the leaves and dark brown edges. In the later stage, black small granules (*sporangiogenic discs*) appear in the center of the lesion, and in severe cases, the lesion is contiguous, causing the leaves to curl, dry up, and fall off [9]. The occurrence of *anthracnose* disease in tea plant leads to a significant decrease in fresh leaf yield and serious deterioration in quality. This disease has occurred in tea growing areas across China, especially in susceptible varieties such as Longjing 43 (LJ43), which may result in a reduction of up to 20% in yield. The *anthracnose* disease of tea plant poses a continuous threat to the economic benefits of tea farmers and the safety of tea production.

The diversity of *anthracnose* pathogens in tea producing areas of our country is complex, with over ten species of *Colletotrichum* identified. Among them, *Colletotrichum camelliae* and *fructicola* are the dominant pathogens; *Colletotrichum camelliae* belongs to the *Colletotrichum anthracnose* complex and has strong pathogenicity to tea leaves. The affected leaves form irregular gray brown disease spots, and later black conidia discs appear on the surface of the disease spots. When the disease spreads, it causes a large number of fallen leaves, significantly affecting tea yield and quality [9]. At present, the prevention and control of tea *anthracnose* mainly rely on chemical pesticides. Although it can control the disease in the short term, long-term use can easily lead to increased resistance of fungal pathogens, environmental pollution, and excessive pesticide residues in tea leaves, which contradicts the concept of sustainable agricultural development. Therefore, exploring the genetic resources of tea plants for disease resistance and cultivating broad-spectrum disease resistant new varieties have become fundamental strategies for achieving safe tea production and sustainable industrial development.

The *R* gene in plants plays an important role in responding to pathogen invasion, participating in the formation of the plant immune signaling network system. Among them, the most concerned are NLR disease resistant genes, whose encoded products specifically recognize effector proteins secreted by pathogens, triggering effector triggered immune responses (ETI) [10]. The structural characteristics of NLR proteins have extremely high similarity. The NBS domain located in the center has conserved motifs such as P-loop, which is the binding site of ADP/ATP and is considered as a molecular switch to activate plant immune response. The LRR domain formed by a series of leucine repeat tandem at the C-terminus is a ligand recognition site and has the function of regulating NLR activity. The variable domain located at the N-terminus is the basis for distinguishing different subgroups [11]. The Toll/Interleukin-1 Receptor (TIR) domain at the N-terminus of TNL proteins exhibits NAD + hydrolase activity, while the N-terminus of CNL proteins has a typical Coil Coil (CC) domain, which has been found to assemble into disease resistance bodies to activate downstream immune responses. RNL proteins are generally considered atypical CNL proteins, and NRG1 in *Arabidopsis* serves as a helper NLR to transmit signals in the form of Ca^2+^ channels to perform programmed cell death [12].

With the completion of a large number of plant whole genome sequencing, the *NLR* gene family has been widely identified and analyzed in different plants, such as *Arabidopsis*, wheat, *Populus tomentosa*, and cassava. The development of gene cloning technology has confirmed that multiple members of the *NLR* gene family play important roles in plant disease resistance. Yr9, located in a highly conserved *NLR* gene cluster in wheat, encodes a typical CC-NBS-LRR (NLR) disease resistant protein that significantly enhances wheat’s resistance to *Puccinia striiformis* f. sp. tritici (Pst) [13]. The *TIR-NBS-LRR* gene *GmTNL16* found in soybean plays an important role in regulating the immune response of soybean to Phytophthora root rot. Evidence shows that its expression is induced by salicylic acid (SA) and jasmonic acid (JA), indicating that it may participate in the immune response through the SA and JA signaling pathways [14].

In recent years, studies have gradually revealed that *NLR* resistant genes play a key regulatory role in the resistance of tea plant to *anthracnose*. The member CsJAZ11 of the tea plant JAZ protein family synergistically regulates the expression of the NLR disease resistance gene CsRPM1 through its interacting protein CsMYC2.2, positively regulating the resistance of tea plant to *anthracnose* pathogen [15]. The interaction between CsNAC17 of the NAM protein family and the transcription factor *CsbHLH62* activates the *NBS-LRR* gene *CsRPM1*, triggering HR related cell death and accumulation of H2O2 around the gloeosporioides infection site, enhancing the resistance of tea plants to *anthracnose* [16]. In 2016, the transcriptome differences between the *anthracnose* resistant variety ZC108 of tea plant and its susceptible parent LJ43 were compared through microarray analysis [17]. Ultimately, 27 candidate genes were identified, including MADS box transcription factors, NLR resistant protein genes, and key enzyme genes in the phenylpropanoid metabolism pathway. Due to the complexity of the tea plant genome, there is still a lack of systematic research on the identification, classification, evolutionary patterns, and expression regulatory networks, especially under *anthracnose* stress, of its members across the entire genome. Therefore, this study aims to conduct whole genome identification, bioinformatics analysis, and expression profiling of the *NBS-LRR* gene family in tea plants. Fully identify the NLR members in the tea plant genome through bioinformatics methods, and analyze their systematic evolution, gene structure, conserved motifs, chromosome localization, and collinearity relationships; Furthermore, qRT-PCR technology was used to investigate the expression dynamics of *NBS-LRR* genes at different time points under *Colletotrichum camelliae* infection, in order to screen key candidate genes involved in the anti *anthracnose* process. The results of this study will provide theoretical basis for further elucidating the molecular mechanism of tea plant resistance to *anthracnose*, and provide valuable genetic resources for molecular breeding of tea plant resistance to *anthracnose*.

## 2. Materials and Methods

### 2.1. Plant Materials and Growth Conditions

The tea cultivars used were the resistant cultivar Sucha 120 (S120) and the susceptible cultivar Ziying 2 (ZY2), planted in the tea garden of Jiangsu Academy of Agricultural Sciences. These plants were placed in a growth chamber (16 h 22 °C, light; 8 h 20 °C, dark daily). Healthy, uniformly growing 3-year-old potted tea seedlings were inoculated with *Colletotrichum* (*Colletotrichum camelliae*) or potato glucose broth medium (PDB). Stress treatments were inoculated with *Colletotrichum*, and plants sprayed with PDB served as controls. *Anthracnose* species were isolated from diseased leaves in the field by tissue isolation method, a highly virulent *anthracnose* strain previously identified by our research group was first cultured on potato dextrose agar (PDA) plates containing 28 °C for 7 days, and then transferred to PDB for 3 days under shaking conditions at 28 °C and 150 rpm. Subsequently, the spore suspension was diluted to 2.0 × 10^5^ spores·mL^−1^ and used to inoculate mature leaves of healthy tea trees. The leaves injected with the pathogen were collected 1, 2, and 4 days after inoculation, frozen in liquid nitrogen, and stored in a −80 °C freezer for subsequent experiments. Conducting three biological repeat experiments and calculating the standard error of the average of three replicates.

### 2.2. Identification, Physicochemical Property Analysis, and Subcellular Localization Prediction of the CsNLR Gene Family

From Tea Plant Information Archive (TPIA) (http://tpia.teaplants.cn) Download “shuchazao_2. genome. fas. gz” (Shuchazao 2 as reference genome) as genome data [18] and retrieve it from PFAM (http://pfam.xfam.org/) Obtain hidden Markov models (HMMs) for NB-ARC (Pfam ID: PF00931) and LRR (Pfam ID: PF00560), and use “Simple HMM search” in TBtools to screen [19], remove duplicates, select candidate members with a critical value of e^−5^, and then further use NCBI’s conservative domain database (CDD) (https://www.ncbi.nlm.nih.gov/Structure/cdd/wrpsb.cgi (accessed on 30 May 2025)) And SMART (https://smart.embl.de/smart/change_mode.cgi (accessed on 29 June 2025)) Verify and ultimately determine the family members of CsNLR. Use the “Protein Paramter Clac” in TBtools v1.120 software to analyze the physicochemical properties of CsNLR, including the calculation of amino acid quantity, relative molecular weight, and isoelectric point physicochemical parameters. Using DeepLoc (https://services.healthtech.dtu.dk/services/DeepLoc-2.1/ (accessed on 5 June 2025)) Perform subcellular localization prediction.

### 2.3. Chromosome Localization and Co Linear Analysis of CsNLR Gene Family

From TPIA (http://tpia.teaplants.cn) Download “shuchazao-v2.gff3.gz” to filter and obtain chromosome length information and gene location information, and use Gene Location Visualize in TBtools to visualize chromosome location information and remove Contig that is not located on the chromosome. Use the “One Step MCScanX” feature in TBtools software to obtain intraspecific collinearity information of CsNLR, and then use “Advanced Circos” to plot it. From Ensemble Plants (https://plants.ensembl.org/index.html (accessed on 10 June 2025)) Download “*Arabidopsis thaliana*.TAIL10.DNA.toplevel.fa.gz” and “*Arabidopsis thaliana*.TAIR10.61.gff3.gz” to obtain the genome and annotation files of *Arabidopsis thaliana*. Download the genome files and annotation files of *Populus trichocarpa* from NCBI under the names “GCF-00002775.5Pl.trichocarpa-v4.1_genomic.fna” and “GCF-00002775.5Pl.trichocarpa-v4.1_genomic.gff”. The collinearity information between species is obtained by “One Step MCScanX” and finally plotted using “Dual Systeny Plot”.

### 2.4. Construction of Phylogenetic Tree

Extract the NB-ARC conserved domain sequences of tea tree CsNLR and *Arabidopsis* NLR proteins, and perform multi sequence alignment using ClustalW. Construct a phylogenetic tree using the Neighbor Joining (NJ) method in MEGA 11 software; The evolutionary distance adopts the Jones Taylor Thornton (JTT) matrix model, and the rate heterogeneity between loci adopts the Gamma distribution (+G), taking into account the invariant loci (+I), i.e., the JTT+G+I model. Use bootstrap test (1000 repetitions) to evaluate the support of each branch node, and display branches with bootstrap values ≥ 50. Finally, CsNLR subgroups were divided based on the phylogenetic tree. 48 NLR proteins in *Arabidopsis* were selected from representative NLR sequences with clear classification that have been published for subfamily classification reference.

### 2.5. Structural Analysis and Motif Prediction of the CsNLR Gene Family

The gene structure information of *CsNLR* was obtained from the genome annotation file “shuchazao_2. gff3. gz”, and the Motif information was obtained from MEME (https://meme-suite.org/meme/index.html (accessed on 11 June 2025)) Conservative structural domain information is obtained from NCBI’s CDD (https://www.ncbi.nlm.nih.gov/Structure/cdd/wrpsb.cgi/ (accessed on 13 June 2025)) Obtained. Finally, the graph was drawn using the “Gene Structure View” feature in TBtools software.

### 2.6. CsNLR Promoter Cis Acting Element Analysis

Using GXF Sequence Extract in TBtools to extract the 2000 bp sequence upstream of the CsNLR start codon. Through the online analysis software PlantCARE (https://bioinformatics.psb.ugent.be/webtools/plantcare/html/ (accessed on 19 June 2025)) Predict and analyze the cis acting elements in the promoter region. The subsequent data visualization is completed through R language (version 4.5.1; https://www.R-project.org/).

### 2.7. Tissue Expression Analysis of CsNLR

From TPIA (http://tpia.teaplants.cn) The expression levels of *CsNLR* in eight tissues including Apical bud, Flower, Fruit, Young leaf, Mature leaf, Old leaf, Root, and Stem were downloaded from the database. Use the “Heatmap” in TBtools v1.120 software to draw a graph.

### 2.8. RNA Extraction and Real Time Fluorescent Quantitative PCR

Based on the results of transcriptome differential expression analysis, 12 significantly differentially expressed genes were selected from 158 identified *CsNLR* genes for qRT-PCR analysis, primer sequences are shown in Table 1. Before amplification, the amplifiability of the cDNA template was preliminarily evaluated based on the amplification status of the internal reference gene glyceraldehyde-3-phosphate dehydrogenase (GAPDH). The amplification procedure is: pre denaturation at 95 °C for 30 s; followed by 40 cycles, each cycle including denaturation at 95 °C for 5 s and annealing/extension at 60 °C for 30 s. After amplification, melting curve analysis is performed to evaluate the specificity of the amplified product. Each group of samples is set with 3 biological replicates, and each biological replicate is set with 3 technical replicates. Total RNA was extracted using a universal plant total RNA rapid extraction kit (centrifugal column type) purchased from Bio Teke (Beijing, China), and an M5 Sprint qRT-PCR kit with gDNA remover purchased from Mei5 Biotechnology (Beijing, China) was used for reverse transcription of cDNA. The qRT-PCR kit ChamQ Blue Universal SYBR qRT-PCR Master Mix was purchased from Vazyme (Nanjing, China). Using LightCycler ^®^ 480 (Roche Diagnostics International Ltd., Rotkreuz, Switzerland) real-time fluorescence quantitative PCR experiments were conducted, and specific primers were designed using Beacon Designer 7 based on the CDS sequence of CsNLR. The reference gene was GAPDH, and the relative expression level of the gene was determined by 2^−ΔΔCT^. The data processing was completed using Excel, the significance analysis of differences was calculated using the R package rstatix (https://www.R-project.org/) and the histogram of relative gene expression levels was drawn using Graphpad Pism9.5.

### 2.9. Predicted Protein Association Network Analysis

Interaction network analysis of 158 CsNLR proteins using STRING12.0 (https://cn.string-db.org/) Completed, beautified and illustrated using Cytoscape v3.8.2. Selecting *Arabidopsis thaliana* as the reference organism and map the tea tree protein sequence to homologous proteins in *Arabidopsis thaliana*; Set confidence score ≥ 0.7 (high confidence); enabled evidence channels: adjacent genes, gene fusion, co-expression, text mining, homologous protein evidence; close the evidence of protein co localization.

## 3. Results and Analysis

### 3.1. Identification and Physicochemical Properties Analysis of CsNLR Gene Family

In this study, we performed HMMsearch based on the hidden Markov models of the NB-ARC domain (Pfam ID: PF00931) and LRR domain (Pfam ID: PF00560) downloaded from PFAM, and ultimately obtained 158 NBS-LRRs, named CsNLR1-CsNLR158 based on their chromosomal localization. According to the analysis of physicochemical properties (Table 2), the protein lengths encoded by 158 CsNLR genes range from 324–1685 amino acids, with isoelectric points (PI values) between 5.4–9.02 and molecular weights between 36.99–193.23 kDa. Fourteen proteins have an instability index less than 40, indicating relative stability, whereas the remaining 144 proteins have an Index greater than 40, suggesting potential instability. The Aliphatic Index ranges from 88.67 to 112.36. The Grand Average of Hydropathicity of 158 *CsNLR* genes is all less than zero, indicating that their hydrophilicity may be higher. The subcellular localization of these 158 proteins was predicted through the DeepLoc-2.1 online analysis website, and the results showed that 34 were distributed in the Endoplasmic reticulum, 57 in the Cell membrane, 25 in Nucleus, 18 in Cytoplasma, and 24 in Lysosome/Vacuum.

### 3.2. Chromosome Localization and Collinearity Analysis of CsNLR Gene Family

**Figure 1:**
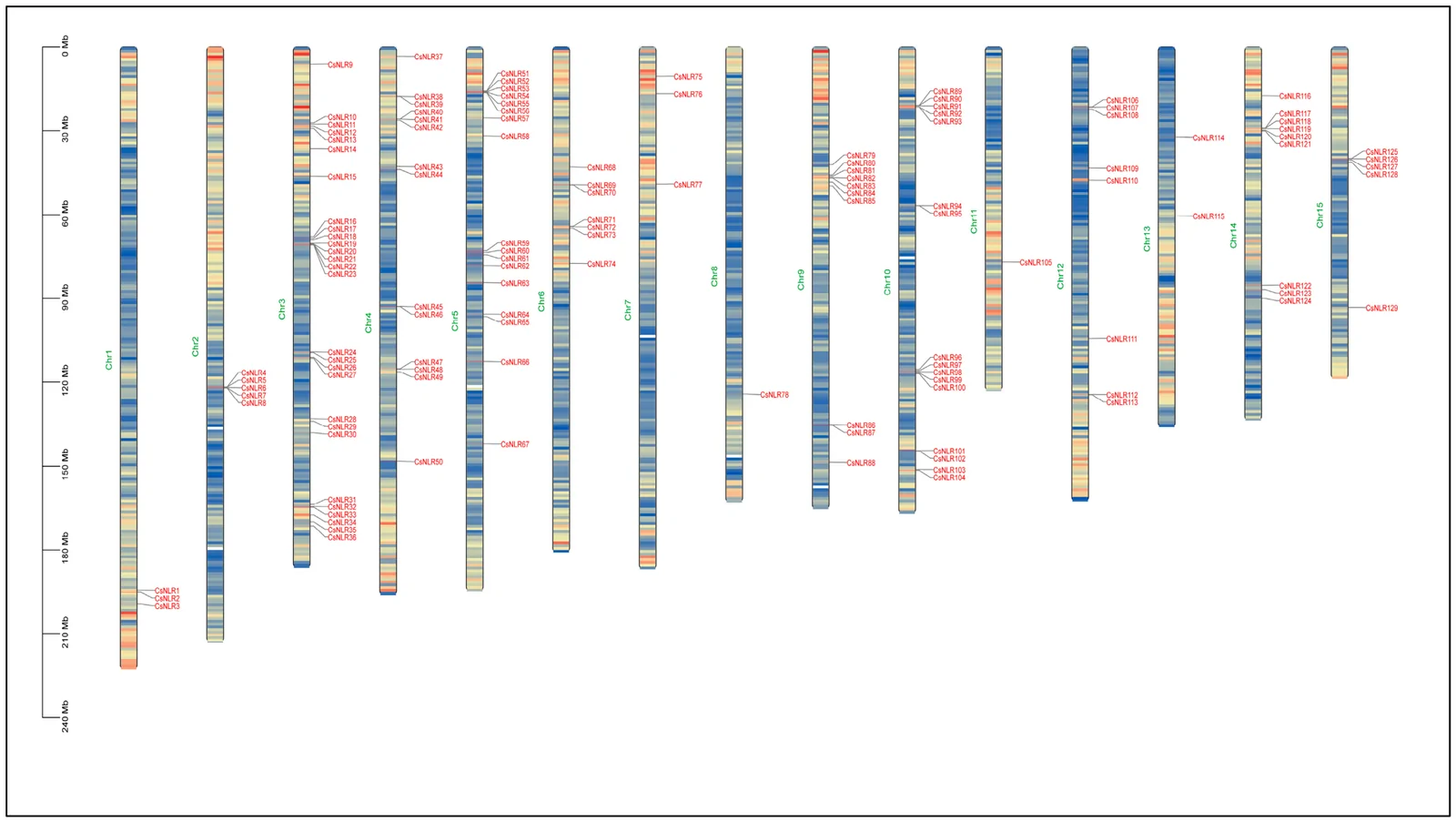
Chromosome Localization of *CsNLR* Gene Family. A total of 129 *CsNLR* genes were mapped to the 15 tea plant chromosomes. Chromosome identifiers are shown beside each chromosome, and gene names are indicated in red, with connecting lines marking their genomic positions. The scale on the left indicates physical positions in megabases (Mb).

To investigate the distribution of *CsNLR* gene family on chromosomes, we used TBtools to visualize chromosome localization. The results showed (Figure 1) that among the 158 *CsNLR* genes, 29 genes were located on gene scaffolds without anchored chromosomes, while the remaining 129 genes were unevenly distributed on the 15 chromosomes of tea plant. The *CsNLR* gene is the most highly represented on chromosome 3, with a total of 28 genes, while there is only one gene on chromosomes 8 and 11. Among these 129 genes, 21 genes form 5 gene clusters distributed on chromosomes 2, 3, 5, 10, and 14. In addition, there are 28 genes located adjacent to each other in pairs on chromosomes 4, 6, 9, 10, 12, and 15.

**Figure 2:**
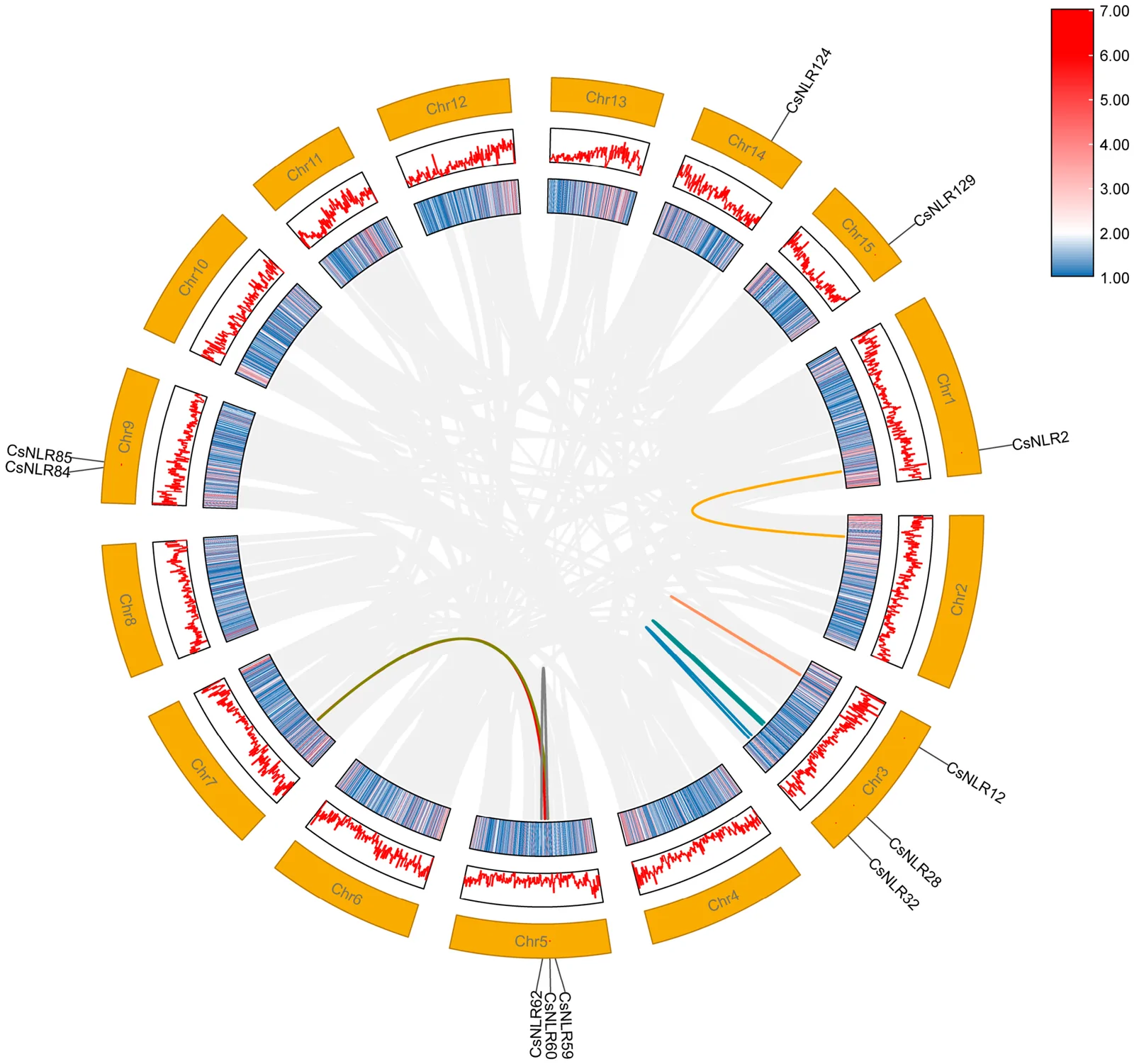
Intraspecific collinearity analysis of *CsNLR* in tea plants. The outer yellow segments represent the 15 tea plant chromosomes. Grey lines indicate genome-wide syntenic relationships, whereas coloured lines highlight syntenic links involving *CsNLR* genes. The corresponding *CsNLR* genes are labelled outside the circle.

**Figure 3:**
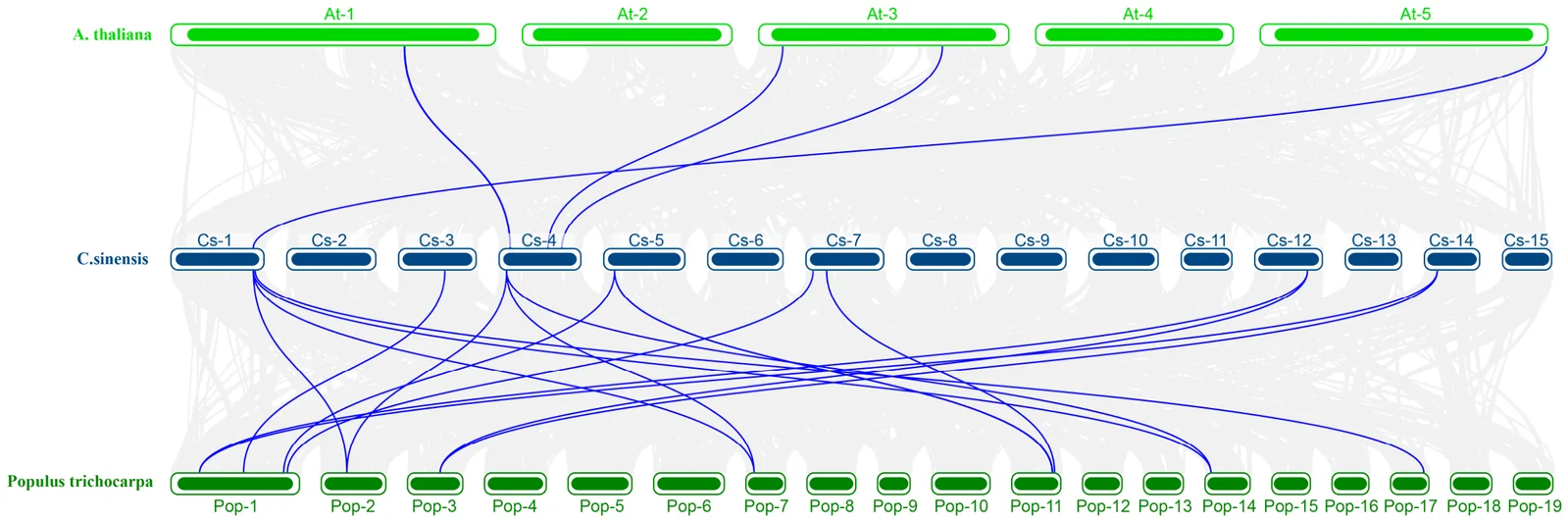
NLR interspecific collinearity analysis of tea trees with *Arabidopsis* and poplar trees. Chromosomes of *Arabidopsis thaliana*, *Camellia sinensis* and *Populus trichocarpa* are arranged from top to bottom. Grey lines indicate genome-wide syntenic relationships between species, whereas blue lines highlight syntenic relationships involving *NLR* genes. Four syntenic gene pairs were identified between *Camellia sinensis* and *Arabidopsis thaliana*, and 16 between *Camellia sinensis* and *Populus trichocarpa*. Chromosome identifiers are shown beside the corresponding chromosomes.

The intra species collinearity analysis of tea plant (Figure 2) identified 11 genes involved in fragment replication events, 4 genes (36.4%) formed collinear pairs with genes in the Contig region that were not located on the chromosome, and 7 genes (63.6%) were directly associated with functional genes outside the family, this result indicates that there is collinearity between some CsNLR fragment replication events and the genomic regions where non NLR gene family are located. The inter species collinearity analysis of tea plant with *Arabidopsis* and poplar plant (Figure 3) showed that 4 pairs of collinearity were identified between CsNLR and AtNLR, while 16 pairs of collinearity were identified between CsNLR and PopNLR. This collinearity and gene replication pattern provide clues for subsequent selection stress analysis.

### 3.3. Structural Analysis and Motif Prediction of CsNLR Gene Family

**Figure 4:**
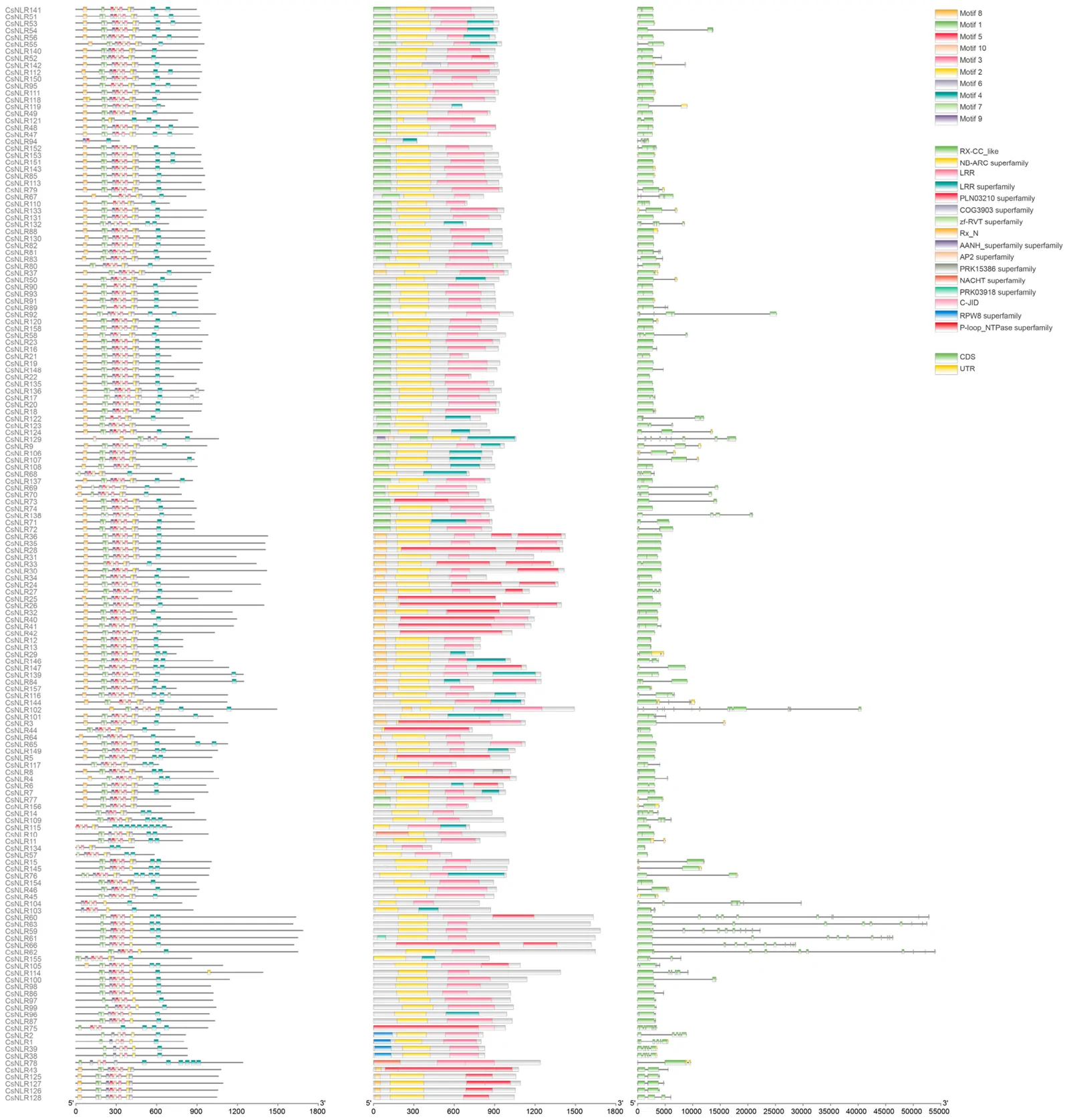
Conserved motifs, domains, and structural analysis of the *CsNLR* gene family. The left panel shows the distribution of 10 conserved motifs identified in CsNLR proteins using MEME, with differently coloured boxes representing individual motifs. The middle panel displays protein domain architectures, with colours indicating the domain annotations shown in the legend. The right panel illustrates gene structures, with green boxes representing coding sequences (CDSs), yellow boxes representing untranslated regions (UTRs), and connecting lines representing introns. Each row corresponds to the same CsNLR member across all three panels. Positions are indicated in amino acids for the motif and domain panels and in base pairs for the gene structure panel.

The analysis of *CsNLR* gene structure (Figure 4) shows that there are differences in the number and arrangement of exons/introns among members of different subfamilies. 20 CsNLRs have no introns, while the number of introns in the other 138 CsNLRs ranges from 1 to 14, and only 42 CsNLRs contain UTRs. This indicates that the functional differences of gene members in different subfamilies are closely related to the complexity of their structures.

To further investigate the functional characteristics of the *CsNLR* gene, we performed Motif prediction on it. Using MEME v5.4.1 software, we predicted 10 conserved Motif sequences among 158 CsNLR protein sequences. The composition and ordering of internal motifs have certain similarities, with a total of 95 CsNLR proteins arranged in the order of Motif 8-1-7-9-5-10-3-2-6-4, of which 92 are distributed in Group II and 3 are in Group I. Among the 30 members of Group IV, only CsNLR105 contains Motif8. 10 CsNLRs lost Motif4, and 5 of them belong to Group III. These Motif proteins are distributed differently in different subfamilies of CsNLR proteins, suggesting that they may play different roles in protein function and interactions.

An analysis was conducted on 158 conserved domains of CsNLR, and a total of 16 domains were identified, including NB-ARC superfamily, LRR, and LRR superfamily, which are typical features of *NLR* genes. In addition, there are also conserved domains RX-CC_like and Rx-N at the N-terminus of *CNL* genes, as well as the conserved domain RPW8 superfamily at the N-terminus of *RNL* genes. We found that although some *CsNLR* genes lack the NB-ARC or LRR superfamily, they share a common feature of having the PLN03210 superfamily. Without identifying the TIR domain, we speculate that structural fragment loss occurred during the long evolutionary process, which may be the result of dynamic genome adjustments.

### 3.4. Classification and Phylogenetic Analysis of CsNLR Gene Family

**Figure 5:**
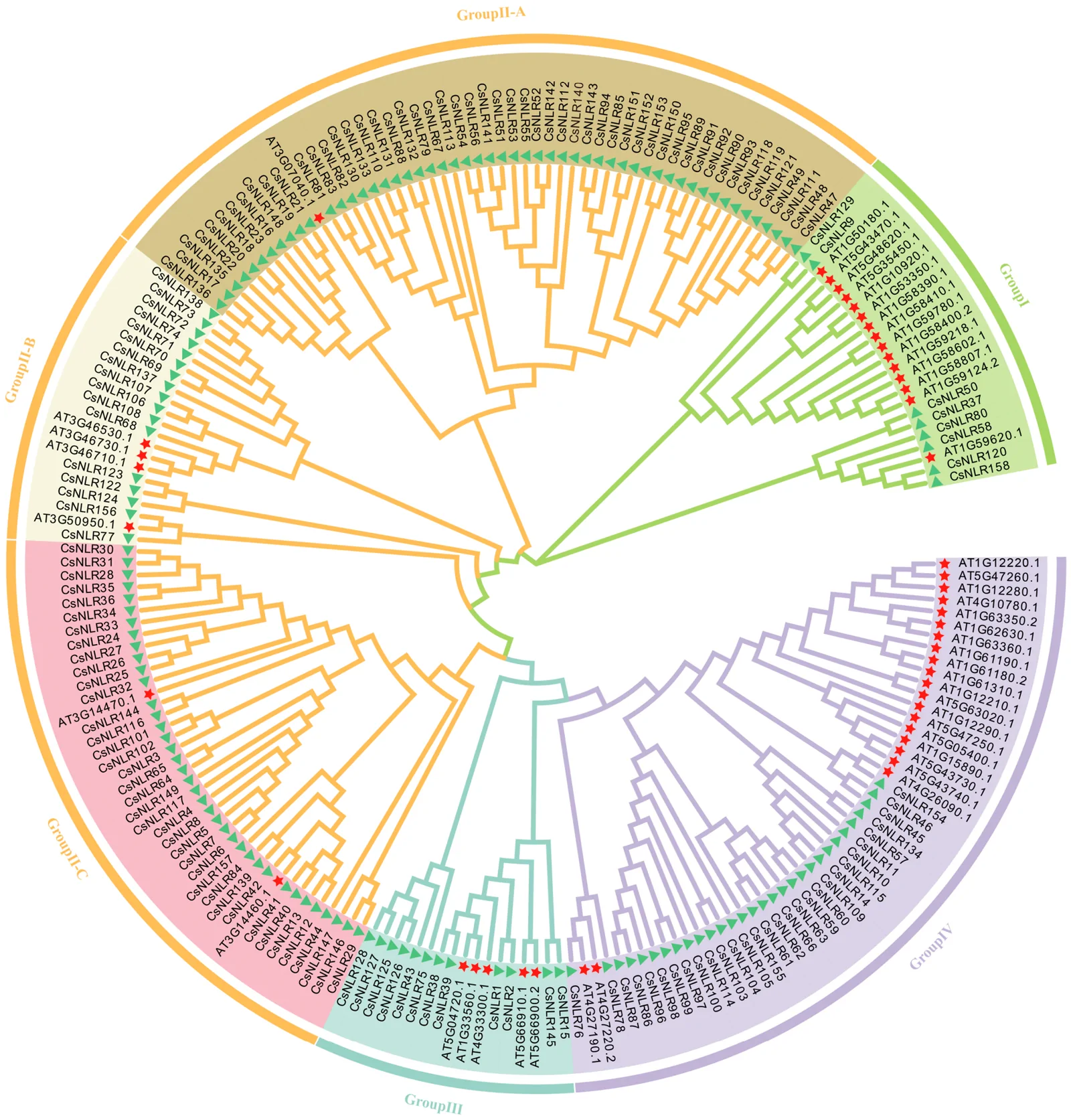
Phylogenetic analysis of NLR proteins in tea trees and *Arabidopsis thaliana*. Green triangles and red stars indicate NLR proteins from *Camellia sinensis* and *Arabidopsis thaliana*, respectively. The proteins were classified into four major groups (Groups I–IV), with Group II further divided into three subgroups (II-A, II-B and II-C). Coloured branches and shaded sectors indicate the corresponding groups and subgroups.

To investigate the evolutionary relationship and classification of the NBS-LRR gene family in tea plants, this study constructed a phylogenetic tree based on 158 CsNLR proteins and 48 AtNLR proteins using the neighbor-joining (NJ) method (Figure 5). According to the sequence and structural characteristics of the protein, it is divided into four subgroups, namely Group I-Group IV, where Group II is divided into Group II-A, Group II-B, and Group II-C. Within the same subgroup, the NBS-LRR proteins of tea and *Arabidopsis* are closely clustered, indicating that they have high homology in evolution, they are closely related in evolution and have the potential for functional conservation, which needs to be verified by subsequent functional experiments. Phylogenetic analysis showed that members of the NBS-LRR family in tea plants were unevenly distributed among these four subgroups, with most members of the *Arabidopsis* NLR family being classified into Group I and Group IV. Group I and Group III have relatively few tea plant members, with 8 and 12 respectively, while Group IV contains 30 tea plant members and 21 *Arabidopsis thaliana* members, indicating that this subgroup holds an important position in both plants. Group II is the largest subgroup, containing 108 CsNLR members, while *Arabidopsis* only has 7 NLR members. It is worth noting that all five gene clusters located on chromosomes belong to Group II, and four of them belong to Group II-A. This indicates that this subgroup has undergone significant gene expansion in tea plant, and this lineage specific expansion pattern strongly suggests that members of this subfamily occupy a central position in the adaptive evolution of tea plant. The evolutionary analysis results provide important theoretical basis and classification framework for further in-depth research on the functions of *NBS-LRR* genes in different subgroups of tea plant. Establishing Group II as the focus of this study and delving into its structural characteristics, replication mechanisms, and expression regulation patterns is crucial for revealing the formation of specific disease resistance mechanisms in tea plants.

### 3.5. Analysis of Cis Acting Elements of CsNLR Gene Family

**Figure 6:**
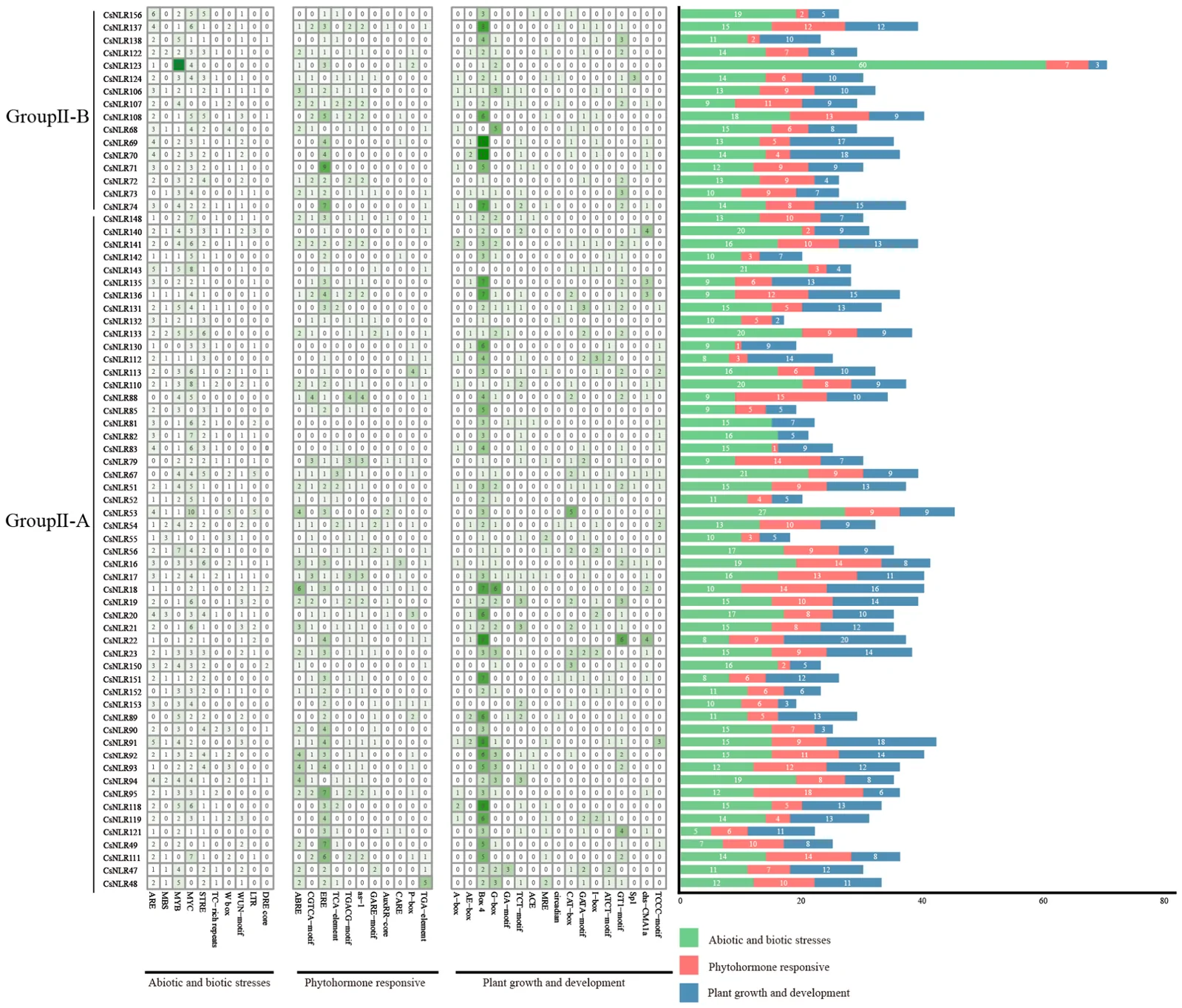
Analysis of cis acting elements in the *CsNLR* gene family. The heatmaps show the number of predicted sites for each element type in each gene, with darker green shading indicating higher counts. Elements are grouped into three functional categories: abiotic and biotic stress responses, phytohormone responses, and plant growth and development. The stacked bars on the right summarize the total number of predicted sites in each category for each gene, represented by green, red and blue, respectively.

The statistical analysis of cis acting elements showed (Supplementary Figure S1) that a total of 38 cis acting elements were identified in the upstream region of the promoter of 158 *CsNLR* genes, which can be divided into three categories. The first type is non biological and biological stress response elements, which bind to specific transcription factors to activate defense related gene expression and enhance plant stress resistance, including ARE, MBS, MYB, MYC, STRE, TC rich repeats, W box, WUN motif, LTR, and DRE core. 149 CsNLRs have MYB binding sites, and 146 CsNLRs have MYC binding sites. In addition, WRKY transcription factor binding sites (W boxes) appeared 80 times. These results indicate the presence of potential binding sites for transcription factors MYB/MYC and WRKY on the *CsNLR* gene. The second type is plant hormone response elements, which mainly respond to plant hormones and regulate hormone mediated physiological processes by binding to specific transcription factors, including ABRE, CGTCA−motif, ERE, TCA−element, TGACG−motif, as−1, GARE−motif, AuxRR−core, CARE, P−box And TGA element. Among them, 95 CsNLRs have abscisic acid responsive elements (ABRE), and 124 CsNLRs have ethylene responsive elements (ERE), suggesting that the ethylene and abscisic acid signaling pathways may be widely involved in transcriptional regulation of this vast gene family. The third category is plant growth and development related components, including A−box, AE−box, Box 4, G−box, GA−motif, TCT−motif, ACE, MRE, circadian, CAT−box, GATA−motif, I−box, ATCT−motif, GT1−motif, Sp1, chs−CMA1a And TCCC motif. The core photoresponsive element Box 4 is distributed among 141 CsNLRs, indicating that the expression of CsNLRs is not only induced by pathogenic stress, but may also be precisely regulated by growth and development signals such as light, hormones, and circadian rhythms. It is worth noting (Figure 6) that multiple key stress response elements (such as ARE, DRE core, TC rich repeats) and plant hormone response elements (such as ABRE, ERE, MBS/MYC) frequently appear in the promoter regions of *CsNLR* genes in Group II-A and Group II-B. The characteristics of CsNLR promoter elements in Group II-A and II-B suggest that this subgroup of genes may be involved in immune signal response, and this hypothesis still needs further in vitro and in vivo experiments to verify. In summary, it is speculated that CsNLR is involved in the stress response, hormone response, growth and development, and light response of tea plants.

### 3.6. Tissue Expression Analysis of CsNLR Gene Family

**Figure 7:**
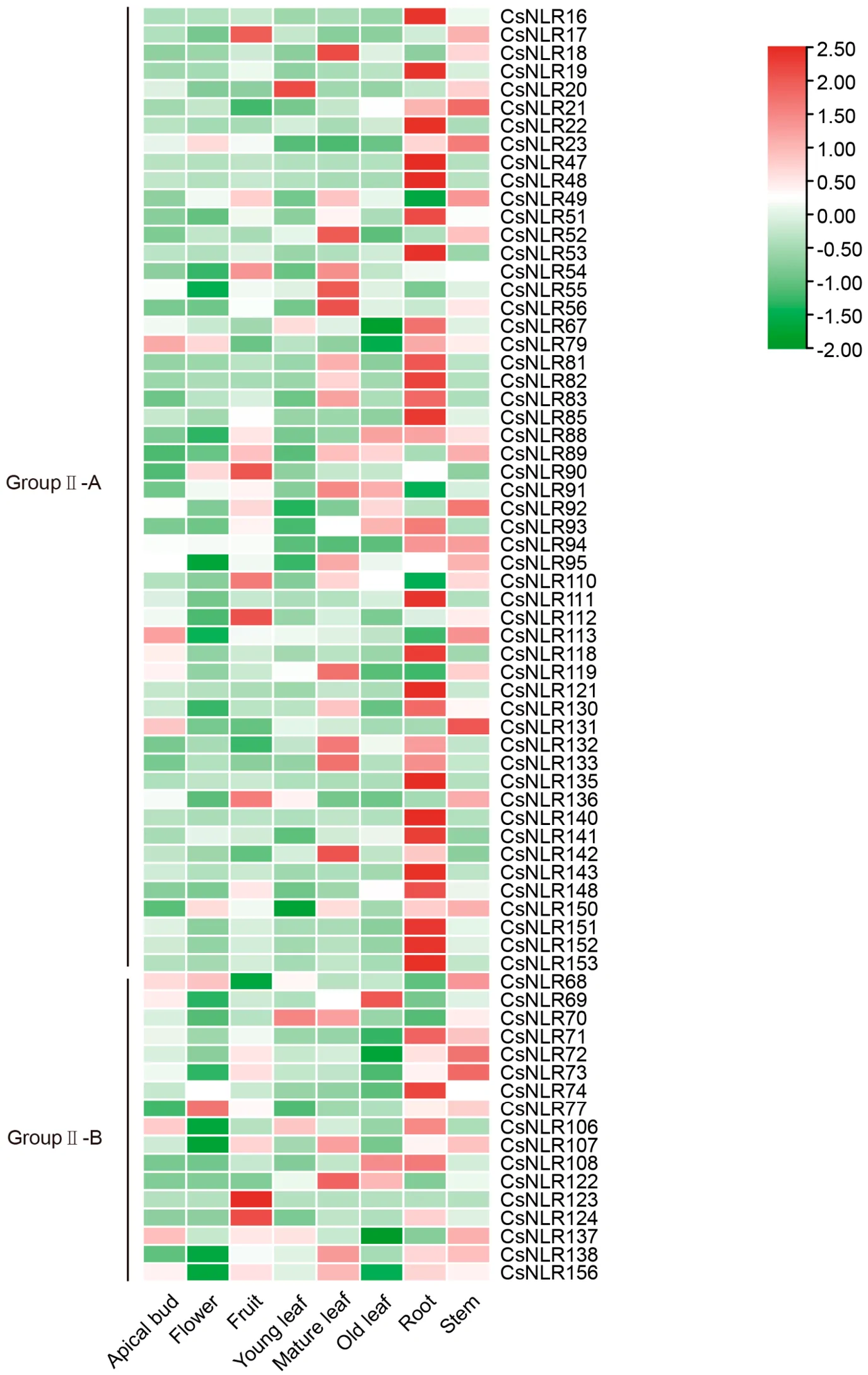
Analysis of *CsNLR* expression patterns in different tissues. The heatmap shows the expression profiles of *CsNLR* genes in subgroups II-A and II-B across eight tissues: apical buds, flowers, fruits, young leaves, mature leaves, old leaves, roots and stems. Expression data were obtained from the Tea Plant Information Archive (TPIA). Rows represent individual *CsNLR* genes, and columns represent tissues. Red and green indicate higher and lower values, respectively, on the expression scale shown. Subgroup assignments are indicated on the left.

The expression patterns of the *CsNLR* gene in different tissues were analyzed, and the results showed (Supplementary Figure S2) that members of the *CsNLR* gene family generally had higher expression levels in Stem, Mature Leaf, and Root, while their expression levels were generally lower in Flower and Apical Bud. A small number of genes were specifically overexpressed in Fruit, Old Leaf, and Young Leaf. Figure 7 shows that the expression levels of CsNLR90, CsNLR132, CsNLR70, CsNLR122, and CsNLR123 are low in all tissues in Group II-A and Group II-B, and may only be expressed at specific developmental stages or when induced by specific external signals. The expression levels of CsNLR67, CsNLR79, CsNLR89, and CsNLR119 in Mature Leaf were significantly higher than those in Young Leaf or Old Leaf, suggesting that they may play an important role in tea plants’ response to *Colletotrichum camelliae* infection. *CsNLR106* maintains high expression levels in multiple tea tree tissues and belongs to a constitutive high expression gene, this expression feature is only a clue to the expression pattern and cannot yet prove its core biological function. Further functional verification is needed.

### 3.7. Expression Pattern Analysis of CsNLR Gene Under Anthracnose camelliae Stress in Tea Plant

**Figure 8:**
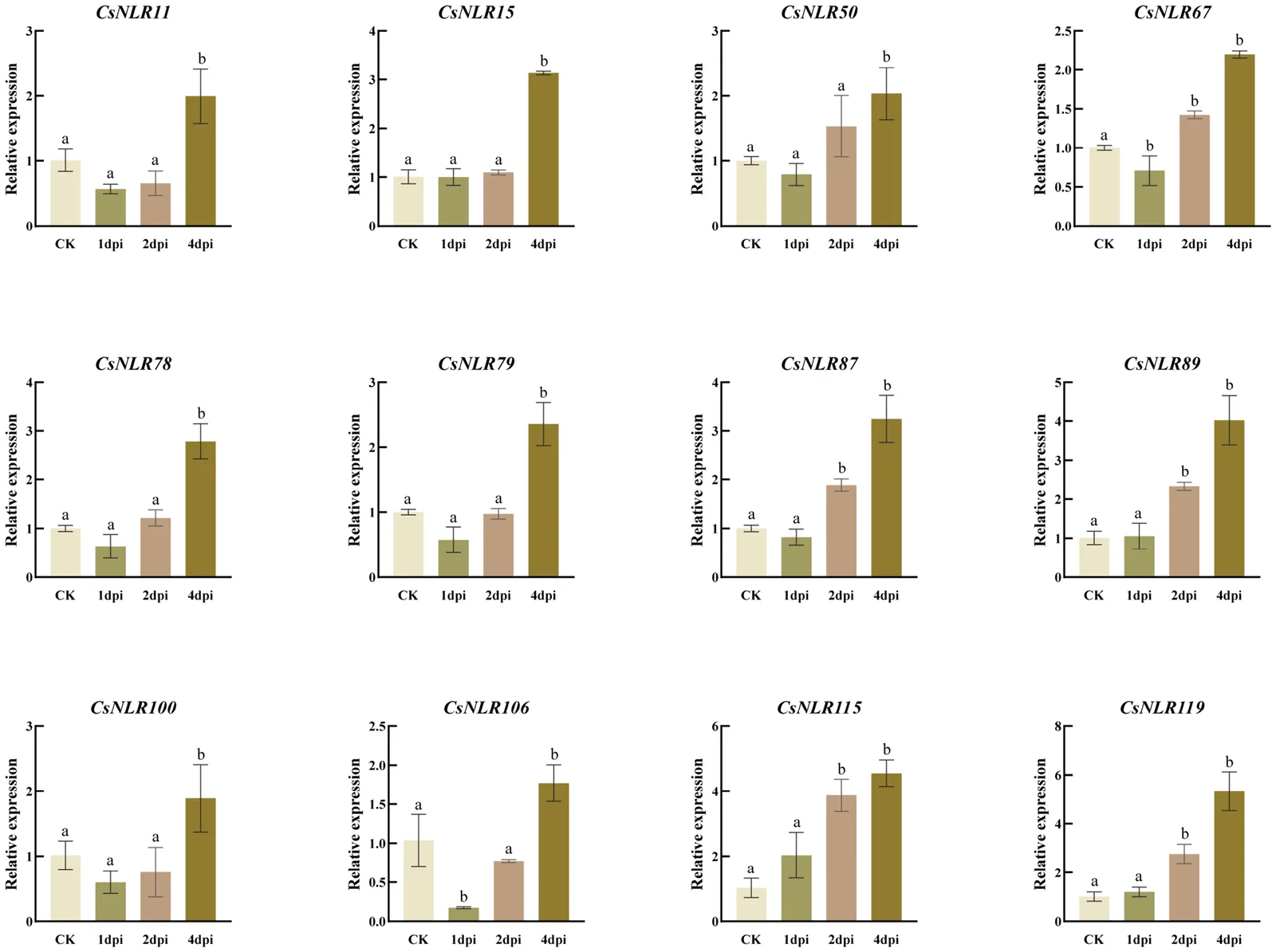
qRT-PCR analysis of *CsNLR* after *anthracnose* infection in Sucha 120. The expression levels of 12 candidate *CsNLR* genes were measured in Sucha 120 at 1, 2 and 4 days after inoculation with *Colletotrichum camelliae*. Uninoculated tea leaves served as the control (CK). Relative expression levels were calculated using the 2^−ΔΔCT^ method, with *GAPDH* as the reference gene and CK as the calibrator. Data are presented as the mean ± SD of three biological replicates. Each post-inoculation time point was compared statistically with CK. a: indicates no significant difference from CK, b: indicates a significant difference from CK. dpi: days post-inoculation. Error bars Standard Deviation (SD).

In order to further verify the expression pattern of *CsNLR* gene in tea plant under *anthracnose* stress, qRT-PCR was used to detect the expression levels of *CsNLR* on the 1st, 2nd, and 4th days after inoculation with *anthracnose* pathogen in the susceptible variety Sucha 120 and the resistant variety Ziying 2. As shown in Figure 8, the expression levels of CsNLR67 and CsNLR106 in Sucha 120 decreased after 1 day of inoculation with *anthracnose*, CsNLR11, CsNLR15, CsNLR50, CsNLR78, CsNLR79, CsNLR87, CsNLR89, CsNLR100, CsNLR115, CsNLR119 There were no significant changes observed. After 2 days post-inoculation, CsNLR67, CsNLR87, CsNLR89, CsNLR115, and CsNLR119 showed an upward trend. After 4 days of inoculation, the expression levels of 12 CsNLRs were significantly upregulated and reached their peak.

**Figure 9:**
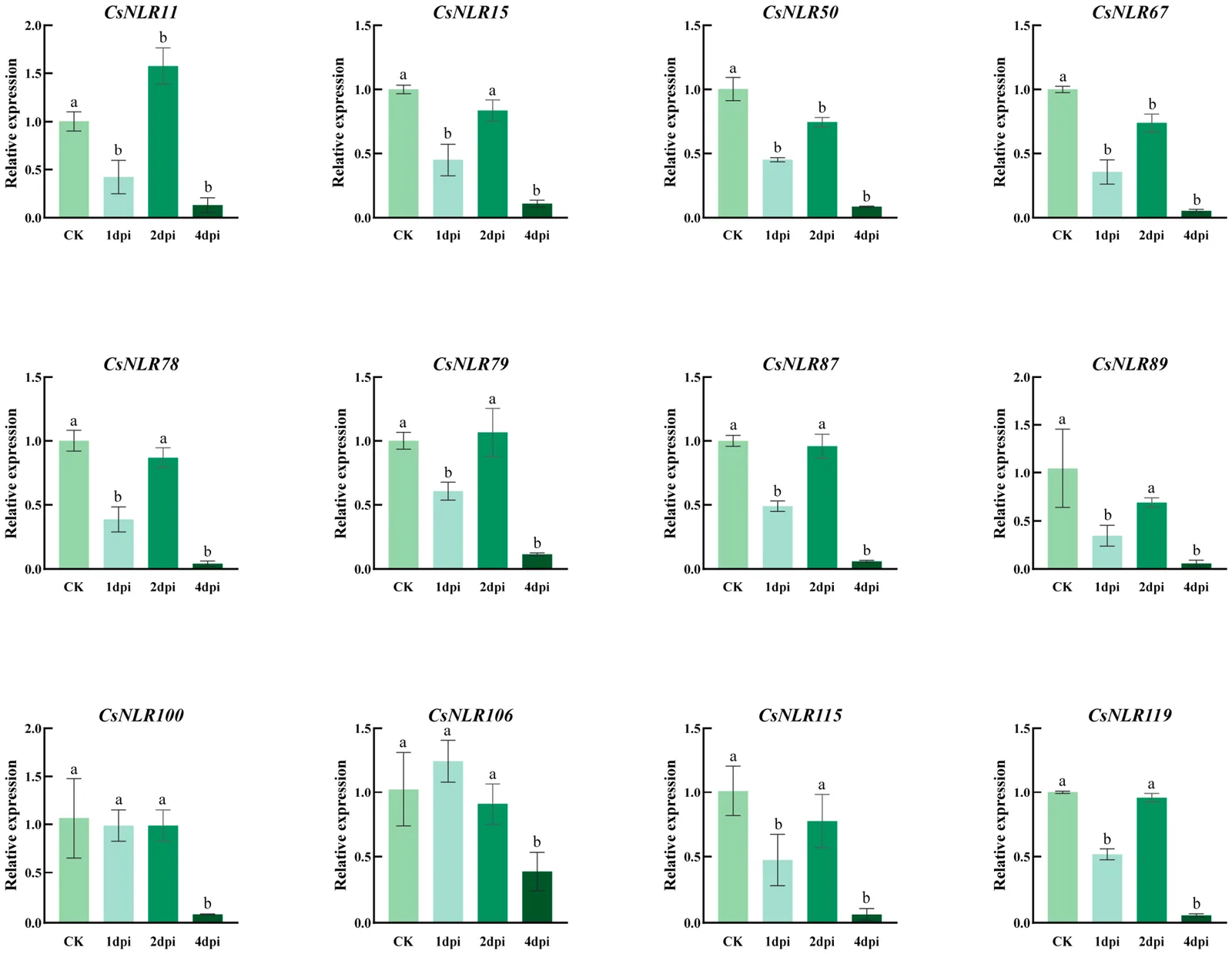
qRT-PCR analysis of *CsNLR* after *anthracnose* infection in Ziying 2. The expression levels of 12 candidate *CsNLR* genes were measured in Ziying 2 at 1, 2 and 4 days after inoculation with *Colletotrichum camelliae*. Uninoculated tea leaves served as the control (CK). Relative expression levels were calculated using the 2^−ΔΔCT^ method, with *GAPDH* as the reference gene and CK as the calibrator. Data are presented as the mean ± SD of three biological replicates. Each post-inoculation time point was compared statistically with CK. a: indicates no significant difference from CK, b: indicates a significant difference from CK. dpi: days post-inoculation. Error bars Standard Deviation (SD).

After inoculation with *anthracnose* bacteria, Ziying 2 showed different trends. As shown in Figure 9, after 1 day of inoculation, except for *CsNLR100* and *CsNLR106*, the expression levels of other *CsNLR* genes were significantly downregulated and rebounded after 2 days of inoculation. It is worth noting that after 4 days of inoculation, all 12 *CsNLR* genes showed extremely significant downregulation. This is completely opposite to the expression trend of *CsNLR* after infection with *anthracnose* in Sucha 120.

### 3.8. Analysis of Predicted Protein Association Network of CsNLR

**Figure 10:**
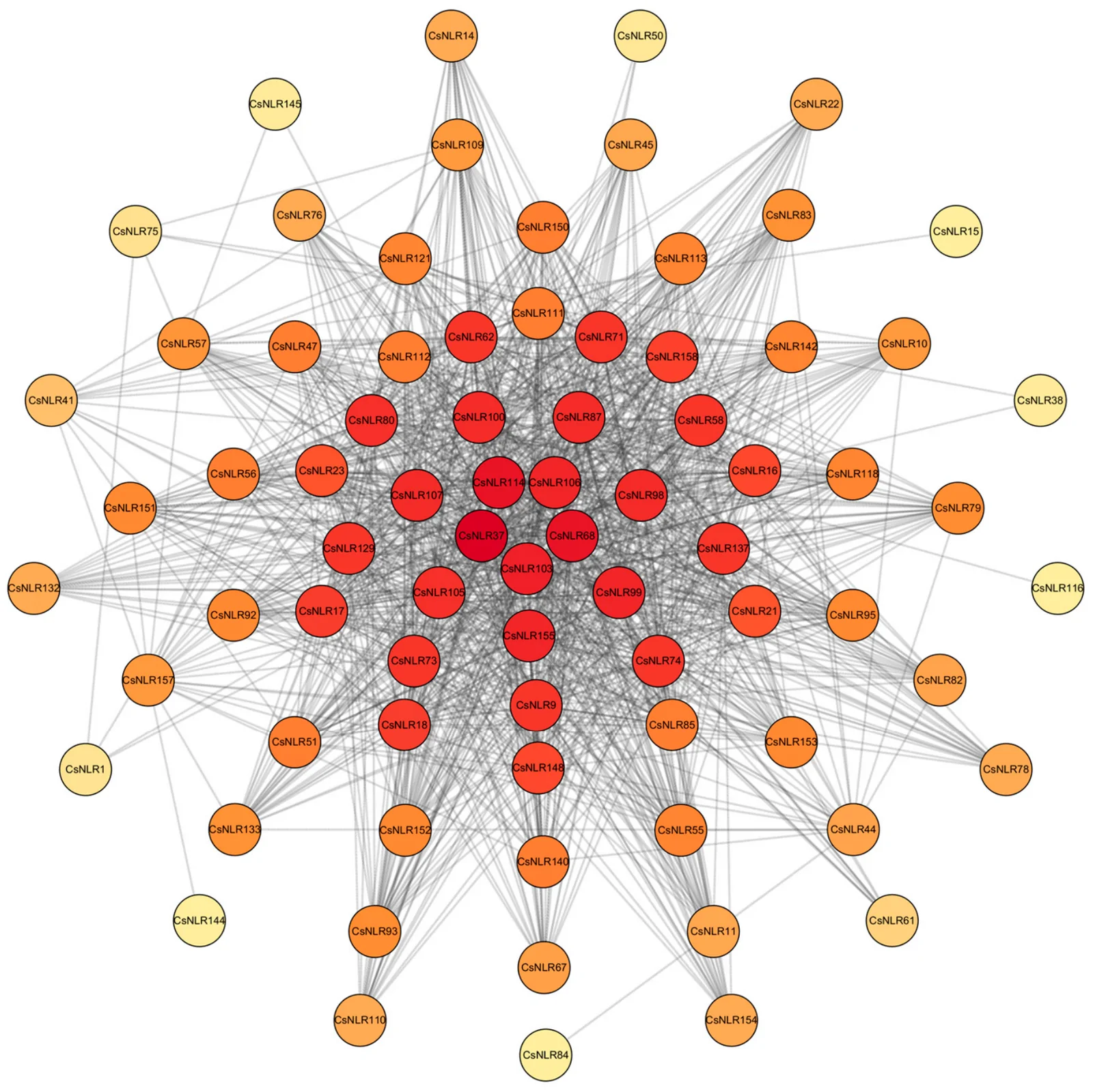
CsNLR predicted protein association network analysis. Nodes represent CsNLR proteins, and connecting lines indicate predicted functional associations between proteins. Protein identifiers are labelled within the nodes. Node colours indicate the degree of connectivity, with darker red representing more connections.

Protein interaction analysis was conducted on 158 CsNLRs using STRING, and the results are shown in Figure 10. A complex predicted protein association network was formed, among them, 77 CsNLR proteins were included in the predictive functional association network, including CsNLR116, CsNLR144, CsNLR15, CsNLR84, CsNLR145, CsNLR38, and CsNLR50, which had fewer protein interactions and may not be core interacting proteins. However, proteins such as CsNLR37, CsNLR68, CsNLR114, and CsNLR103 are highly connected nodes in the predicted protein association network, indicating their potential core association positions. The actual protein interactions require further experimental verification.

## 4. Discussions

### 4.1. Structural and Functional Analysis of CsNLR

According to the Zig-Zag model, pathogens break through the first line of defense of the plant immune system, pattern-triggered immunity (PTI), by secreting specific effector proteins that effector-triggered susceptibility (ETS), while plants specifically recognize these effectors or their modifications to host targets through intracellular or membrane resistance proteins, thereby activating stronger and faster effector-triggered immunity (ETI) [20]. In the defense network of plant immunity, *R* genes play a crucial role, and NLR class *R* genes are the most abundant among the discovered *R* genes [21,22].

In this study, a sequence search strategy based on Hidden Markov Model (HMM) was adopted to identify members of the NLR gene family in tea plant. Hidden Markov Models of NB-ARC domain (Pfam ID: PF00931) and LRR domain (Pfam ID: PF00560) were downloaded from the PFAM database and HMMsearch was performed to screen 158 *CsNLR* gene family members. The analysis of physicochemical properties shows that all 158 CsNLR proteins are hydrophilic, and the predicted subcellular localization distribution is Endoplasmic reticulum, Cell membrane, Nucleus, Cytoplasm, Lysosome, Vacuole in China, its localization exhibits a high degree of heterogeneity, which is highly consistent with its function as an intracellular immune receptor. The diversity of localization suggests that they may monitor different pathogen effectors in their respective microenvironments, like “sentinels” deployed at key cellular sites, collectively forming a multi-level and three-dimensional intracellular immune monitoring network [23]. CsNLR is unevenly distributed on chromosomes, although it is distributed on each chromosome, it forms distinct clusters on chromosomes 3 and 5. To investigate their causes, we conducted intraspecific collinearity analysis, which showed that these clusters are closely related to multiple gene replication events. Perhaps these replication events are not only the key reasons for the expansion of gene numbers, but also provide a genetic material basis for the diversification of disease resistance specificity [24]. Based on amino acid sequence based phylogenetic analysis, 158 CsNLRs were divided into four subgroups. It is noteworthy that no TIR domains were found in any of the four subgroups. The 18 sequences downloaded from the HMM model (Pfam ID: PF01582) for TIR were found to have missing LRR (Toll/Interleukin-1 Receptor) domains. Partial domain deletions were also observed in the TNL or CNL subgroups of cassava [25,26]. Some studies have found that despite the absence of LRR and other structural domains, certain TIR only proteins such as RBA1 and TX14 can still exercise immune function through their TIR domains. It retains the complete enzyme active center, possesses NADase (nicotinamide adenine dinucleotide enzyme) activity and ADP ribosyltransferase activity, and more notably, this type of TIR protein can induce phase separation to form higher-order assemblies (agglomerations), thereby activating its enzyme activity and immune function [27]. Among the 158 CsNLRs, 117 members (74.1%) have two conserved domains at the N-terminus, RX-CC_like or Rx-N, and the linear arrangement order of their conserved motifs also shows a high degree of consistency, indirectly reflecting that CsNLR family members may be highly conserved in immune signaling or pathogen recognition.

### 4.2. CsNLR May Be Involved in the Defense Process of Tea Plant Against Anthracnose

Through the analysis of cis acting elements in the *NLR* gene family of tea plants, it was found that most members have cis acting elements related to disease resistance or plant hormone response, which can be bound by corresponding transcription factors. These transcription factors trigger different metabolic pathways through activation/inactivation, thereby causing plants to produce defensive proteins, hormones, or other metabolites [28,29]. The enrichment of MYB binding sites and W boxes in the promoter region of *CsNLR* gene suggests that MYB protein may be involved in stress response and metabolic regulation in tea plants [30]. In addition, it may integrate multiple signal inputs through *WRKY* transcription factors to finely regulate immune response in different physiological states [31]. The presence of plant hormone responsive elements such as gibberellin responsive elements (GARE motif and P-box) and salicylic acid responsive elements (TCA element) suggests that the expression of CsNLR is controlled by a complex and precise regulatory network, which is dynamically fine tuned by hormone signaling [32]. This coexistence phenomenon is not accidental, which may mean that the promoter activity of these genes can be differentially regulated based on the invasion of specific pathogens.

*Anthracnose* is one of the main fungal diseases of tea leaves [33]. The antagonistic variety ZC108 and susceptible variety LJ43 were inoculated with *C. gloeosporiides*, and it was found that the susceptibility of ZC108 was significantly lower than that of LJ43 [34]. A study has found that RNA-Seq was performed on the resistant variety ZC108 and susceptible variety LJ43 inoculated with *C. fructicola*. ZC108 screened for more differentially expressed genes, and the number of NLR resistant genes was also higher. Significant hypersensitivity (HR) response and H2O2 accumulation were observed around the infection site [35]. However, the differences in expression levels and quantities of *NLR* genes cannot serve as direct evidence that disease resistant varieties are more resistant. A study has found that the *LOV1* gene in *Arabidopsis* encodes a typical CNL disease resistance protein, which can rapidly induce the expression of salicylic acid (SA)—dependent defense marker gene *PR-1* and the synthesis of antibacterial substances. However, activation by specific pathogen toxins can erroneously lead to cell death, endowing plants with pathogenicity [36]. This is like the front and back of a coin.

Purple tea leaves often have a higher anthocyanin content than green leaves [37]. The biosynthetic pathway of anthocyanins is strongly correlated with the activation of tea plant defense signaling pathways. In addition, anthocyanins, as powerful natural antioxidants, can effectively eliminate the accumulation of reactive oxygen species caused by pathogen infection [38]. This constitutes the physiological basis for the strong disease resistance of purple tea plant varieties. A study has found that after inoculating tea leaves with *anthracnose* bacteria, a “pink ring” is formed around the lesion. The main component of the pink ring is anthocyanin-3-O-galactoside, which enhances resistance to *anthracnose* [39].

In order to investigate the expression patterns of *CsNLR* gene family under *anthracnose* stress, this study selected susceptible variety Sucha 120 and resistant variety Ziying 2 to inoculate *Colletotrichum camelliae*. The expression changes of *CsNLR* gene were detected by qRT-PCR on days 1, 2, and 4 after inoculation, and the expression changes of *CsNLR* gene were different in the two varieties. This phenomenon may be explained by by the Zig-Zag model [20]. In the early stages of infection, pathogen associated molecular patterns (PAMPs) are recognized by pattern recognition receptors (PRRs) to stimulate plant defense responses, with PTI dominating. At this time, *CsNLR* shows a brief downward trend in resistant and susceptible varieties. When the second layer of immune response ETI of plants is activated by R protein, the expression of *CsNLR* in the resistant variety Ziying 2 reached a peak after the second day of inoculation. After finishing the precise recognition of pathogen effectors, there was a significant decline on the the fourth day after inoculation, marking the completion of immune response. On the contrary, in the susceptible variety Sucha 120, the expression of *CsNLR* showed an increasing trend on the first, second, and fourth days after inoculation, and reached the peak on the the fourth day, which reflected the disorder of defense signals caused by the lack of accurate identification of pathogen effectors, which led to the formation of susceptible phenotype [40]. In summary, based on the expression patterns of the five *CsNLR* genes *CsNLR67*, *CsNLR79*, *CsNLR89*, *CsNLR106*, and *CsNLR119* under *anthracnose* stress in the susceptible variety Sucha 120 and the resistant variety Ziying 2, it can be inferred that they are involved in the regulation of tea *anthracnose* disease.

### 4.3. Research Significance, Limitations and Future Research Prospects

Previous studies on tea plant disease resistance have mainly focused on physiological and biochemical responses under *anthracnose* stress, transcriptome-wide differential gene screening, and the functional verification of a small number of disease-resistant genes. Existing research has confirmed that *NLR* gene family are core resistance genes involved in plant immune response and pathogen recognition in multiple plant species, and several disease resistance-related regulatory pathways in tea plants have been preliminarily identified. However, the systematic identification, structural characteristics, and evolutionary traits of the *NLR* gene family in tea plants remain unsystematic. Most importantly, the expression variation and regulatory network of tea *NLR* genes in response to *anthracnose* infection in different tea germplasms are still unclear. There is a lack of targeted clarification on the functional differences of *NLR* genes in resistant and susceptible tea varieties, which severely restricts the in-depth exploration of the molecular mechanism of tea plant resistance to *anthracnose* and the mining of core resistance genes, forming a key knowledge gap in the field of tea disease resistance molecular biology.

This study effectively compensates for the above research deficiencies. Based on the latest Shuchazao2 tea reference genome, we systematically identified 158 *CsNLR* gene family and comprehensively characterized their physicochemical properties, chromosomal distribution, phylogenetic relationships, gene structure, and stress-responsive cis-acting elements, which supplemented the basic systematic data of the tea *NLR* gene family that was previously incomplete. Furthermore, combined with qRT-PCR verification of two distinct tea varieties (anthracnose-resistant common tea variety Sucha 120 and purple tea variety Ziying 2), this study clarified the differential expression patterns of *CsNLR* genes under *anthracnose* infection, and revealed the opposite expression trend of key *CsNLR* genes in different germplasms at 4 days after inoculation. Meanwhile, the core node regulatory role and 77 CsNLR proteins were included in the predictive functional association network, and there were differences in connectivity among different members. These findings fill the research blank of systematic identification and *anthracnose* response functional analysis of tea *NLR* gene family, clarify the potential differential resistance mechanism of *CsNLR* genes in different tea germplasms, and provide reliable candidate genes and theoretical support for the subsequent exploration of tea *anthracnose* resistance molecular mechanism and disease-resistant germplasm screening.

Nevertheless, this study still has certain inherent limitations. Firstly, the current conclusion is only based on bioinformatics analysis and transcriptional expression verification at the RNA level. The protein expression characteristics, post-translational modification modes, and specific binding modes of *CsNLR* genes with downstream target proteins under *anthracnose* stress have not been verified. Secondly, this study only detected the gene expression changes at a single key time point (4 days after inoculation), and the dynamic expression pattern of *CsNLR* genes in the whole process of *anthracnose* infection and resistance response remains unclear. In addition, the disease resistance function of *CsNLR* genes was only inferred through expression trends and network correlation analysis, and the specific resistance mechanism differences of *CsNLR* genes caused by germplasm variation have not been verified by functional experiments.

Future research can further expand the time-series sampling of *anthracnose* infection to construct the dynamic expression regulatory model of *CsNLR* genes throughout the infection process. On this basis, gene overexpression, gene silencing and transgenic functional verification experiments can be carried out to clarify the in-vivo disease resistance function of core *CsNLR* genes. Moreover, multi-omics combined analysis and protein interaction verification assays can be used to screen upstream regulatory factors and downstream interactive target genes of *CsNLRs*, further elucidating the complete molecular regulatory network of *CsNLR* genes mediating tea plant *anthracnose* resistance. In addition, expanding the test germplasm resources can verify the universal adaptability of the differential expression mechanism of *CsNLR* genes in different tea varieties, which will further deepen the systematic understanding of the molecular mechanism of tea plant resistance to *anthracnose*.

## Figures and Tables

**Table 1:** Quantitative primer sequence of NLR.

Primer Name	Sequences of Primers (5′–3′)	Purpose
*CsNLR11*-qF	GGTCTGACGAACTTGAGATGGT	RT-qPCR
*CsNLR11*-qR	TGGAGATGGACTCTGGCAGATA	RT-qPCR
*CsNLR15*-qF	TAGTGGGTACAGTGGACAATGC	RT-qPCR
*CsNLR15*-qR	GTTGCATTGGGCCCTTACAAAT	RT-qPCR
*CsNLR50*-qF	AACCTCAAAACCCTGACACTGT	RT-qPCR
*CsNLR50*-qR	ATCCCCCAGCAAGACAATTCAT	RT-qPCR
*CsNLR67*-qF	CAGACGAGGATTCAAGTCCCAA	RT-qPCR
*CsNLR67*-qR	ACAAGGATTTGTTTGGCAGCTC	RT-qPCR
*CsNLR78*-qF	TTGATCTGCCGAACATGAGGAA	RT-qPCR
*CsNLR78*-qR	AGGTTTGTTCTTGTCCCCAACT	RT-qPCR
*CsNLR79*-qF	AAGTTTCAATGGCAGAACTCGC	RT-qPCR
*CsNLR79*-qR	AGGAGCACCCTCAAGATCTAGT	RT-qPCR
*CsNLR87*-qF	CATTGCGATGCACTGGTATGAG	RT-qPCR
*CsNLR87*-qR	AGTTCCTTCATCCCTCCGTAGA	RT-qPCR
*CsNLR89*-qF	AGGCTGGTGGTAGAGAAATTGG	RT-qPCR
*CsNLR89*-qR	CCTGTAATTCAGCAACTCCCCT	RT-qPCR
*CsNLR100*-qF	ATGACGTTGTGATGGCTGTAGT	RT-qPCR
*CsNLR100*-qR	CCTCCCCATATTCCAAACCCAA	RT-qPCR
*CsNLR106*-qF	AAGATGTCCTTCAGAGCTGGTG	RT-qPCR
*CsNLR106*-qR	GGATCAGTTTCCACACAGGGAT	RT-qPCR
*CsNLR115*-qF	CTTCGGGGGAATCACAACCTAA	RT-qPCR
*CsNLR115*-qR	CTTCTTCAATCTGCCTCCCAGT	RT-qPCR
*CsNLR119*-qF	GATGAGCACGATTCGATTGTGG	RT-qPCR
*CsNLR119*-qR	GTAAGCCTCTGCAACTTCCTCT	RT-qPCR
*CsGAPDH*-qF	TTGGCATCGTTGAGGGTCT	RT-qPCR
*CsGAPDH*-qF	CAGTGGGAACACGGAAAGC	RT-qPCR

**Table 2:** Physical and Chemical Properties Analysis and Subcellular Localization Prediction of CsNLR.

Sequence ID	Gene Name	Chr	Size (aa)	Mass (Da)	pI	Instability Index	Aliphatic Index	Grand Averageof Hydropathicity	Subcellular Location
CSS0015606.1	CsNLR1	Chr1	800	90,471.46	5.58	40.04	102.11	−0.104	Endoplasmic reticulum
CSS0039663.1	CsNLR10	Chr3	982	113,003.13	6.03	45.26	97.33	−0.329	Endoplasmic reticulum
CSS0033810.1	CsNLR100	Chr10	1140	129,702.37	5.98	41.72	100.79	−0.192	Cell membrane
CSS0035373.1	CsNLR101	Chr10	1018	116,142.05	5.87	50.19	96.62	−0.259	Cell membrane
CSS0040544.1	CsNLR102	Chr10	1492	171,257.18	8.26	50.15	88.67	−0.404	Nucleus
CSS0022334.1	CsNLR103	Chr10	870	98,309.55	6.46	47.65	108.62	−0.058	Cytoplasm
CSS0033420.1	CsNLR104	Chr10	787	88,646.32	6.54	45.24	106.53	0.005	Cytoplasm
CSS0049903.1	CsNLR105	Chr11	1090	124,222.6	5.56	41.22	99.17	−0.11	Cell membrane
CSS0008112.1	CsNLR106	Chr12	885	100,664.75	8.53	48.91	102.34	−0.198	Endoplasmic reticulum
CSS0010223.1	CsNLR107	Chr12	878	99,962.59	7.32	46.35	99.6	−0.258	Cell membrane
CSS0021741.1	CsNLR108	Chr12	901	10,3618	6.72	45.58	100.06	−0.16	Cytoplasm
CSS0020769.1	CsNLR109	Chr12	965	112,268.35	6.3	51.93	98.13	−0.311	Cytoplasm
CSS0041386.1	CsNLR11	Chr3	792	90,465.94	6.1	41.96	103.7	−0.099	Endoplasmic reticulum
CSS0041004.1	CsNLR110	Chr12	695	79,683.52	8.75	36.21	101.25	−0.302	Cell membrane
CSS0021802.1	CsNLR111	Chr12	928	107,172	7.55	52.26	105.12	−0.222	Cell membrane
CSS0038765.1	CsNLR112	Chr12	934	107,697.65	7.54	46.62	104.25	−0.165	Endoplasmic reticulum
CSS0041462.1	CsNLR113	Chr12	931	107,170.41	8.99	50.51	103.63	−0.228	Lysosome/Vacuole
CSS0047500.1	CsNLR114	Chr13	1389	157,819.66	5.44	47.46	100.66	−0.14	Cell membrane
CSS0043378.1	CsNLR115	Chr13	713	82,686.31	6.73	54.97	106.19	−0.323	Cytoplasm
CSS0013242.1	CsNLR116	Chr14	1126	128,402.31	6.99	45.46	101.99	−0.144	Nucleus
CSS0008076.1	CsNLR117	Chr14	614	69,714.68	7.22	43.54	99.98	−0.134	Endoplasmic reticulum
CSS0046089.1	CsNLR118	Chr14	906	104,223.07	7.97	47.24	109.71	−0.155	Endoplasmic reticulum
CSS0005423.1	CsNLR119	Chr14	659	75,463.27	7.31	46.22	108.25	−0.137	Lysosome/Vacuole
CSS0044235.1	CsNLR12	Chr3	794	89,680.63	6.34	40.84	103.74	−0.202	Endoplasmic reticulum
CSS0029270.1	CsNLR120	Chr14	924	105,165.64	8.67	43.2	104.2	−0.086	Nucleus
CSS0019751.1	CsNLR121	Chr14	755	86,972.36	6.89	49.17	102.38	−0.291	Cell membrane
CSS0002249.1	CsNLR122	Chr14	795	92,045.08	8.67	39.21	97.43	−0.192	Endoplasmic reticulum
CSS0018810.1	CsNLR123	Chr14	842	96,330.64	9.02	45.5	103.22	−0.237	Endoplasmic reticulum
CSS0048866.1	CsNLR124	Chr14	864	99,163.55	8.68	42.48	97.01	−0.236	Cell membrane
CSS0045788.1	CsNLR125	Chr15	1058	119,680.7	6.52	47.55	94.04	−0.211	Nucleus
CSS0043846.1	CsNLR126	Chr15	1054	119,143.98	6.3	48.56	94.87	−0.216	Nucleus
CSS0010973.1	CsNLR127	Chr15	1091	123,458.83	6.16	46.69	94.23	−0.212	Nucleus
CSS0045919.1	CsNLR128	Chr15	1046	117,946.78	5.43	48.31	91.2	−0.23	Nucleus
CSS0045016.1	CsNLR129	Chr15	1059	122,303.2	7.21	43.62	94.2	−0.344	Nucleus
CSS0046154.1	CsNLR13	Chr3	794	89,679.69	6.58	40.73	103.74	−0.202	Endoplasmic reticulum
CSS0049743.1	CsNLR130	Contig111	958	109,251.13	7.8	39.79	104.95	−0.148	Nucleus
CSS0013999.1	CsNLR131	Contig111	945	108,178.26	8.46	44.08	100.66	−0.292	Cell membrane
CSS0034693.1	CsNLR132	Contig136	689	78,825.42	6.87	41.49	103.08	−0.224	Lysosome/Vacuole
CSS0025563.1	CsNLR133	Contig136	969	111,340.16	8.41	40.78	100.87	−0.305	Cell membrane
CSS0002797.1	CsNLR134	Contig219	432	49,612.98	6.62	49.52	112.36	−0.101	Cytoplasm
CSS0000327.1	CsNLR135	Contig324	895	102,705.87	6.78	47.71	98.77	−0.27	Cell membrane
CSS0007355.1	CsNLR136	Contig324	950	108,586.62	8.42	50.3	94.69	−0.299	Lysosome/Vacuole
CSS0012875.1	CsNLR137	Contig454	866	100,341.28	6.09	52.27	97.02	−0.347	Cell membrane
CSS0009198.1	CsNLR138	Contig524	859	100,377.89	8.48	49.09	96.32	−0.376	Cell membrane
CSS0048754.1	CsNLR139	Contig549	1243	140,935.27	7.17	46.4	102.3	−0.146	Cell membrane
CSS0022045.1	CsNLR14	Chr3	881	101,566.58	5.69	47.98	96.35	−0.368	Endoplasmic reticulum
CSS0029392.1	CsNLR140	Contig582	904	103,685.2	7.81	42.22	106.62	−0.132	Cell membrane
CSS0024041.1	CsNLR141	Contig582	895	102,130.41	8.96	43.62	106.63	−0.199	Cell membrane
CSS0001084.1	CsNLR142	Contig582	923	105,468.75	6.18	38.64	103.98	−0.184	Cell membrane
CSS0019885.1	CsNLR143	Contig629	943	108,440.75	8.65	44.4	100.04	−0.229	Cell membrane
CSS0047799.1	CsNLR144	Contig629	1122	126,433.09	6.26	43.88	107.46	0.035	Cytoplasm
CSS0050315.1	CsNLR145	Contig705	993	113,601.1	6.75	42.13	99.63	−0.247	Cytoplasm
CSS0027751.1	CsNLR146	Contig869	1018	11,5454.34	5.54	43.22	101.29	−0.239	Nucleus
CSS0044768.1	CsNLR147	Contig869	1135	129,805.56	5.95	39.15	103.89	−0.239	Nucleus
CSS0028816.1	CsNLR148	Contig917	917	105,833.01	7.93	48.26	101.28	−0.197	Nucleus
CSS0019797.1	CsNLR149	Contig995	1052	119,434.5	5.51	54.66	98.04	−0.216	Nucleus
CSS0015515.1	CsNLR15	Chr3	1006	115,860.84	6.72	43.87	100.93	−0.26	Cytoplasm
CSS0005361.1	CsNLR150	Contig1143	916	105,797.19	8.48	42.33	106.72	−0.148	Cell membrane
CSS0012043.1	CsNLR151	Contig1143	926	106,396.33	8.02	46.34	103.35	−0.227	Cell membrane
CSS0006288.1	CsNLR152	Contig1143	882	100,592.25	8.51	46.82	105.18	−0.076	Nucleus
CSS0029187.1	CsNLR153	Contig1143	930	107,037.82	8.74	47.42	101.33	−0.281	Lysosome/Vacuole
CSS0043699.1	CsNLR154	Contig1191	892	101,209.01	5.63	48.87	102.89	−0.092	Lysosome/Vacuole
CSS0012670.1	CsNLR155	Contig1210	860	97,320.74	6.01	43.99	102.44	−0.225	Cytoplasm
CSS0027506.1	CsNLR156	Contig1219	704	79,825.16	6.65	42.41	98.18	−0.223	Cell membrane
CSS0003647.1	CsNLR158	Contig1294	914	103,902.23	8.88	43.53	104.3	−0.095	Lysosome/Vacuole
CSS0033254.1	CsNLR16	Chr3	928	106,651.1	8.65	50.59	103.97	−0.174	Lysosome/Vacuole
CSS0013256.1	CsNLR17	Chr3	913	104,331.75	8.8	49.71	95.02	−0.32	Lysosome/Vacuole
CSS0044882.1	CsNLR18	Chr3	930	106,888.6	7.52	48.49	97.55	−0.298	Cell membrane
CSS0030052.1	CsNLR19	Chr3	939	107,983.47	7.5	50.9	103.06	−0.15	Lysosome/Vacuole
CSS0019496.1	CsNLR2	Chr1	814	92,039.31	5.67	39.58	99.99	−0.112	Nucleus
CSS0031870.1	CsNLR20	Chr3	937	107,423.03	6.67	49.63	94.95	−0.321	Lysosome/Vacuole
CSS0032389.1	CsNLR21	Chr3	706	80,763.39	8.33	49.08	97.86	−0.206	Lysosome/Vacuole
CSS0035610.1	CsNLR22	Chr3	725	82,952.74	8.8	45.59	94.5	−0.378	Cell membrane
CSS0019632.1	CsNLR23	Chr3	937	108,048.45	7.93	49.49	103.06	−0.169	Lysosome/Vacuole
CSS0002460.1	CsNLR24	Chr3	1371	155,129.91	6.61	45.85	98.81	−0.167	Cell membrane
CSS0004816.1	CsNLR25	Chr3	906	103,526.96	6.56	41.08	95.09	−0.23	Endoplasmic reticulum
CSS0025332.1	CsNLR26	Chr3	1396	158,636.58	5.84	46.43	97.88	−0.185	Endoplasmic reticulum
CSS0040534.1	CsNLR27	Chr3	1158	132,179.81	5.75	48.13	96.04	−0.23	Endoplasmic reticulum
CSS0042866.1	CsNLR28	Chr3	1407	160,325.66	6.7	43.31	97.8	−0.234	Endoplasmic reticulum
CSS0043327.2	CsNLR29	Chr3	744	85,431.3	6.22	43.07	101.14	−0.316	Endoplasmic reticulum
CSS0011964.1	CsNLR3	Chr1	1128	128,824.83	7.13	48.93	107.07	−0.165	Cell membrane
CSS0041398.1	CsNLR30	Chr3	1416	161,326.58	6.66	42.44	98.3	−0.257	Cytoplasm
CSS0013081.1	CsNLR31	Chr3	1190	136,146.05	5.97	44.23	95.99	−0.249	Lysosome/Vacuole
CSS0019855.1	CsNLR32	Chr3	1161	132,812.94	5.57	43.41	101.97	−0.145	Lysosome/Vacuole
CSS0023320.1	CsNLR33	Chr3	1339	152,302.45	5.48	45.57	96.8	−0.238	Lysosome/Vacuole
CSS0009427.1	CsNLR34	Chr3	839	95,928.97	7.97	38.16	94.34	−0.302	Endoplasmic reticulum
CSS0011777.1	CsNLR35	Chr3	1404	160,578.83	6.14	42.53	97.66	−0.249	Nucleus
CSS0047741.1	CsNLR36	Chr3	1424	162,566.81	6.09	44.45	96.97	−0.276	Nucleus
CSS0003263.1	CsNLR37	Chr4	1001	114,155.72	5.8	53.99	95.4	−0.371	Cell membrane
CSS0046943.1	CsNLR38	Chr4	827	94,612.95	5.94	52.44	104.99	−0.105	Cytoplasm
CSS0020808.1	CsNLR39	Chr4	827	94,637.99	6.01	52.53	104.99	−0.105	Cytoplasm
CSS0043086.1	CsNLR4	Chr2	1061	119,850.88	6.66	44.96	99.86	−0.141	Endoplasmic reticulum
CSS0034627.1	CsNLR40	Chr4	1194	135,815.85	6.82	49.34	101.65	−0.204	Cell membrane
CSS0013343.1	CsNLR41	Chr4	1170	133,110.85	6.53	49.96	103.85	−0.187	Cell membrane
CSS0027619.1	CsNLR42	Chr4	1029	117,154.14	6.57	50.24	103.32	−0.192	Endoplasmic reticulum
CSS0016819.1	CsNLR43	Chr4	1077	122,463.54	6.05	48.85	99.99	−0.191	Nucleus
CSS0038611.1	CsNLR44	Chr4	735	84,067.39	6.62	48.41	99.43	−0.187	Endoplasmic reticulum
CSS0001794.1	CsNLR45	Chr4	895	102,840.95	5.79	46.75	101.11	−0.208	Cell membrane
CSS0040253.1	CsNLR46	Chr4	914	104,891.19	5.62	46.5	100.07	−0.221	Cell membrane
CSS0008380.1	CsNLR47	Chr4	867	99,410.15	6.89	48.92	108.37	−0.195	Endoplasmic reticulum
CSS0044705.1	CsNLR48	Chr4	908	104,130.58	7.54	47.2	105.09	−0.211	Endoplasmic reticulum
CSS0048848.1	CsNLR49	Chr4	867	99,738.83	8.63	52.37	107.68	−0.217	Lysosome/Vacuole
CSS0029174.1	CsNLR5	Chr2	1010	114,737.96	5.8	46.24	102.28	−0.133	Cell membrane
CSS0011120.1	CsNLR50	Chr4	933	106,364.03	8.6	48.55	99.88	−0.273	Cell membrane
CSS0046258.1	CsNLR51	Chr5	919	105,101.89	8.85	43.66	107.64	−0.162	Cell membrane
CSS0007394.1	CsNLR52	Chr5	894	101,938.79	6	36.25	107.15	−0.11	Cell membrane
CSS0029059.1	CsNLR53	Chr5	932	106,973.69	7.85	44.66	104.25	−0.198	Cell membrane
CSS0037395.1	CsNLR54	Chr5	922	105,775.03	7.03	42.05	101.79	−0.224	Cell membrane
CSS0020984.1	CsNLR55	Chr5	952	108,838.27	7.81	39.28	106.54	−0.085	Lysosome/Vacuole
CSS0037533.1	CsNLR56	Chr5	905	103,615.74	8.29	41.18	104.21	−0.216	Cell membrane
CSS0042967.1	CsNLR57	Chr5	582	66,913.51	5.88	53.51	106.67	−0.178	Cytoplasm
CSS0034558.1	CsNLR58	Chr5	981	112,815.25	8.07	49.01	99.5	−0.269	Endoplasmic reticulum
CSS0037245.1	CsNLR59	Chr5	1685	193,228.48	5.76	42.73	97.35	−0.276	Cell membrane
CSS0002401.1	CsNLR6	Chr2	965	109,410.29	6.07	47.81	99.17	−0.19	Lysosome/Vacuole
CSS0009386.1	CsNLR60	Chr5	1633	186,650.84	6.06	37.77	101.98	−0.183	Cell membrane
CSS0006875.1	CsNLR61	Chr5	1647	188,071.56	6.44	49.61	98.41	−0.288	Cell membrane
CSS0050384.1	CsNLR62	Chr5	1648	188,634.72	6.48	41.56	99.18	−0.264	Endoplasmic reticulum
CSS0029815.1	CsNLR63	Chr5	1611	184,574.5	6.31	41.51	95.66	−0.333	Cell membrane
CSS0029466.1	CsNLR64	Chr5	882	100,794.3	7.34	48.43	97.89	−0.246	Nucleus
CSS0040834.1	CsNLR65	Chr5	1127	128,091.11	7.53	51.85	102.72	−0.189	Endoplasmic reticulum
CSS0034659.1	CsNLR66	Chr5	1618	184,282.85	5.86	48.42	102.37	−0.223	Cell membrane
CSS0001695.1	CsNLR67	Chr5	818	94,241.05	8.84	45.12	101.49	−0.313	Lysosome/Vacuole
CSS0020118.1	CsNLR68	Chr6	711	82,194.9	6.3	39.65	98.13	−0.145	Cytoplasm
CSS0026473.1	CsNLR69	Chr6	767	89,487.21	7.09	53.33	95.72	−0.335	Cell membrane
CSS0050355.1	CsNLR7	Chr2	981	111,421.81	6.2	43.69	96.85	−0.196	Endoplasmic reticulum
CSS0007634.1	CsNLR70	Chr6	783	91,161.46	6.8	50.37	97.75	−0.288	Nucleus
CSS0047959.1	CsNLR71	Chr6	881	101,844.26	6.33	47.78	96.36	−0.341	Cell membrane
CSS0043037.1	CsNLR72	Chr6	878	101,618.88	6.66	47.22	96.9	−0.39	Nucleus
CSS0036428.1	CsNLR73	Chr6	874	100,866.22	7.19	46.63	95.13	−0.369	Cell membrane
CSS0034815.1	CsNLR74	Chr6	894	103,710.26	5.76	49.08	96.69	−0.33	Endoplasmic reticulum
CSS0035728.1	CsNLR75	Chr7	980	110,862.79	6.09	50.05	96.85	−0.189	Nucleus
CSS0050203.1	CsNLR76	Chr7	986	111,464.65	5.43	44.06	107.66	−0.087	Cytoplasm
CSS0025623.1	CsNLR77	Chr7	876	100,312.26	6.33	51.28	97.9	−0.228	Cell membrane
CSS0044487.1	CsNLR78	Chr8	1239	140,194.97	6.22	43.86	103.93	−0.162	Cytoplasm
CSS0034762.1	CsNLR79	Chr9	957	110,303.42	7.64	45.96	109.75	−0.118	Lysosome/Vacuole
CSS0049384.1	CsNLR8	Chr2	1020	115,213.5	6.02	47.29	101.58	−0.134	Cell membrane
CSS0004319.1	CsNLR80	Chr9	1024	117,570.62	8.91	44.98	97.08	−0.235	Endoplasmic reticulum
CSS0005973.1	CsNLR81	Chr9	999	114,210.41	8.71	43.13	100.46	−0.289	Nucleus
CSS0050158.1	CsNLR82	Chr9	954	108,783.9	6.73	44.03	101.86	−0.212	Endoplasmic reticulum
CSS0040057.1	CsNLR83	Chr9	969	110,712.31	7.99	44.12	104.38	−0.221	Endoplasmic reticulum
CSS0008440.1	CsNLR84	Chr9	1246	141,078.43	7.86	47.12	102.22	−0.141	Cell membrane
CSS0025652.1	CsNLR85	Chr9	956	109,377.88	8.43	47.33	101.55	−0.192	Nucleus
CSS0042836.1	CsNLR86	Chr9	1019	115,328.47	6.09	41	100.24	−0.211	Cell membrane
CSS0004067.1	CsNLR87	Chr9	1031	117,414.67	5.35	40.23	101.04	−0.19	Cell membrane
CSS0001639.1	CsNLR88	Chr9	956	109,681.93	7.01	40.53	105.56	−0.211	Lysosome/Vacuole
CSS0000501.1	CsNLR89	Chr10	905	102,815.8	8.11	50.1	104.15	−0.107	Lysosome/Vacuole
CSS0032454.2	CsNLR9	Chr3	972	109,818.53	8.96	47.66	102.97	−0.15	Endoplasmic reticulum
CSS0047102.1	CsNLR90	Chr10	896	101,932.92	8.7	46.67	104.52	−0.12	Cell membrane
CSS0033798.1	CsNLR91	Chr10	907	103,815.02	7.75	45.94	101.32	−0.131	Cell membrane
CSS0022083.1	CsNLR92	Chr10	1038	117,958.15	8.52	45.8	105.91	−0.057	Endoplasmic reticulum
CSS0022127.1	CsNLR93	Chr10	903	103,019.23	8.66	46.74	104.55	−0.108	Lysosome/Vacuole
CSS0016922.1	CsNLR94	Chr10	324	36,991.2	7.11	47.78	107.96	0.061	Cytoplasm
CSS0048873.1	CsNLR95	Chr10	895	102,709.23	8.47	36.56	108.54	−0.169	Endoplasmic reticulum
CSS0030965.1	CsNLR96	Chr10	990	112,468.4	5.24	41.65	99.32	−0.222	Cell membrane
CSS0026281.1	CsNLR97	Chr10	1017	115,538.51	5.85	40.27	94.6	−0.291	Cell membrane
CSS0045612.1	CsNLR98	Chr10	1000	113,440.55	5.31	41.4	98.94	−0.24	Cell membrane
CSS0029306.1	CsNLR99	Chr10	1040	117,839.36	6.05	38.69	94.95	−0.249	Cell membrane

## Data Availability

Dataset available on request from the authors.

## Supplementary Materials

The following supporting information can be downloaded at: https://www.mdpi.com/article/10.3390/genes17101302/s1, Figure S1: Analysis of 158 CsNLR genes cis acting elements. The heatmaps show the number of predicted sites for each element type in each gene, with darker green shading indicating higher counts. Elements are grouped into three functional categories: abiotic and biotic stress responses, phytohormone responses, and plant growth and development. The stacked bars on the right summarize the total number of predicted sites in each category for each gene, represented by green, red and blue, respectively; Supplementary Figure S2: Expression patterns of 158 CsNLR genes in different tissues. The heatmap shows the expression profiles of 158 CsNLR genes in eight tissues: apical buds, flowers, fruits, young leaves, mature leaves, old leaves, roots and stems. Expression data were obtained from the Tea Plant Information Archive (TPIA). Red and green indicate higher and lower values, respectively, on the expression scale shown.
