# Supplementary material for: Genome-Wide Identification of NLR Gene Family and Expression Patterns in Response to Colletotrichum camelliae Infection in Tea Plant (Camellia sinensis)
Source: Genes (Basel). 2026 Oct 20;17(10):1302. doi: 10.3390/genes17101302 (PMC13704370; doi:10.3390/genes17101302)
Supplement: Supplementary file 1 [file genes-17-01302-s001.zip › genes-4587294-supplementary.pdf]

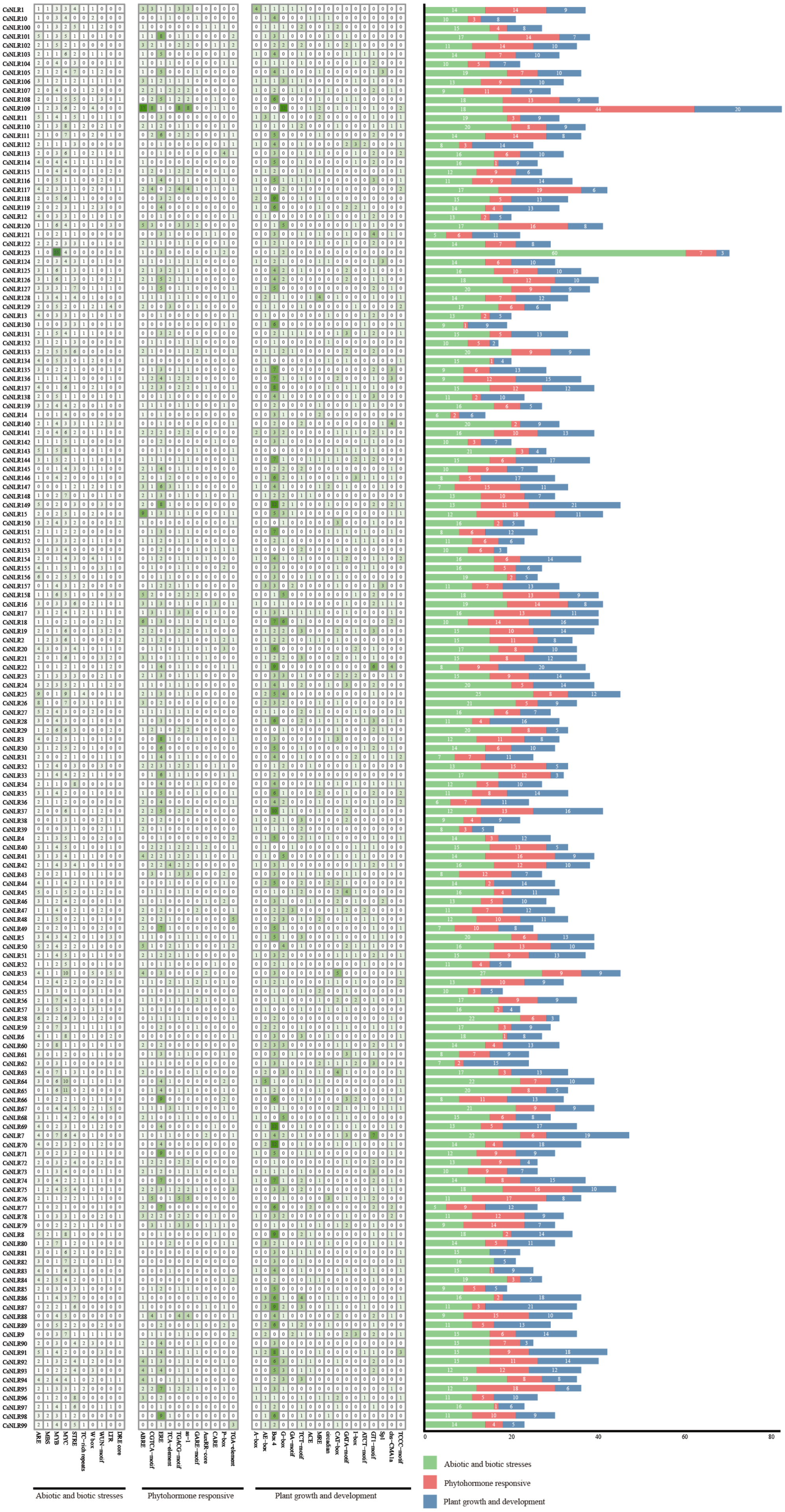
Abiotic and biotic stresses
Phytohormone responsive
Plant growth and development
0
20
40
60
80

Supplementary Figure S1. Analysis of 158 *CsNLR* genes cis acting elements. The heatmaps show the number of predicted sites for each element type in each gene, with darker green shading indicating higher counts. Elements are grouped into three functional categories: abiotic and biotic stress responses, phytohormone responses, and plant growth and development. The stacked bars on the right summarize the total number of predicted sites in each category for each gene, represented by green, red and blue, respectively.

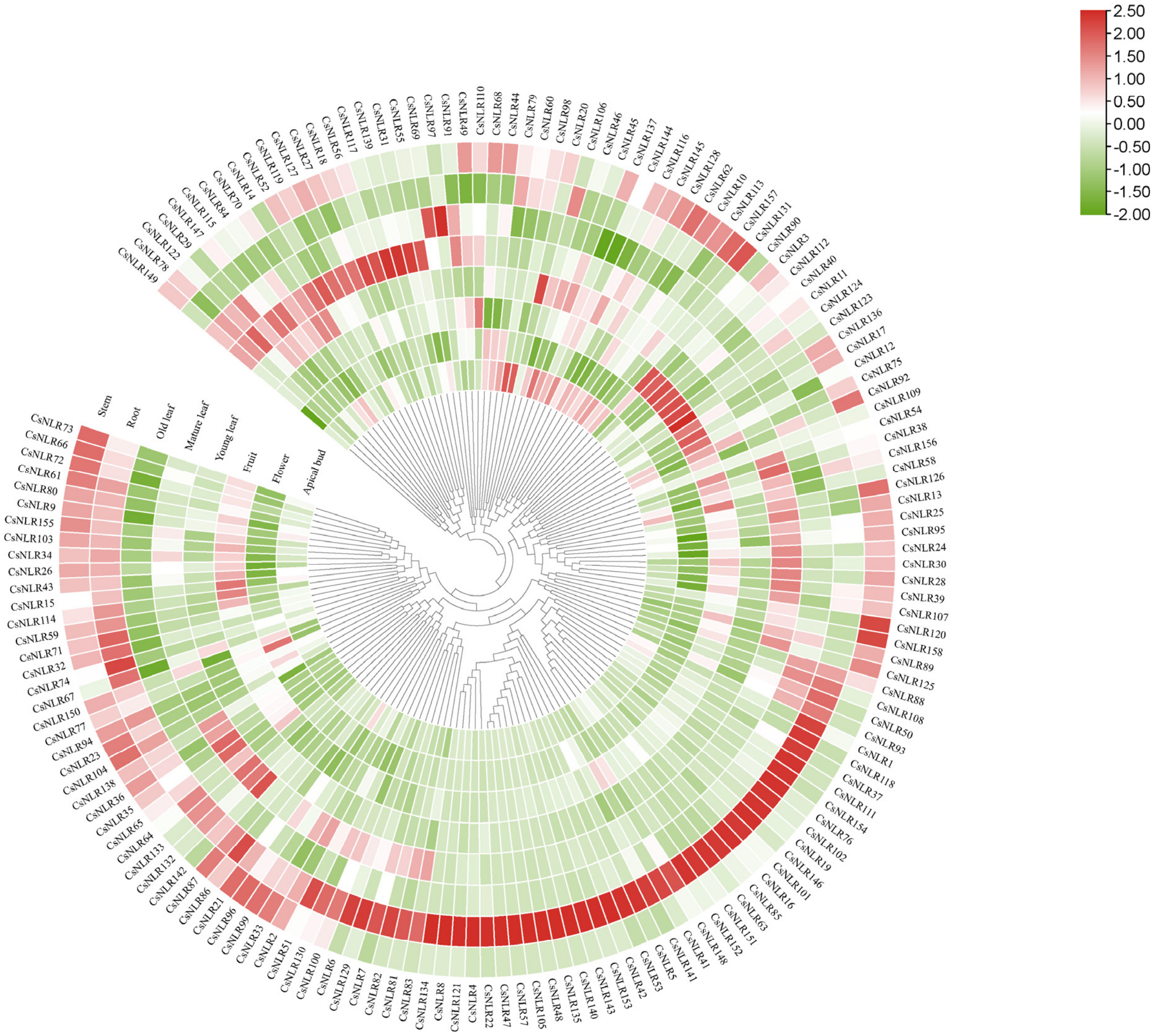

Supplementary Figure S2. Expression patterns of 158 *CsNLR* genes in different tissues. The heatmap shows the expression profiles of 158 *CsNLR* genes in eight tissues: apical buds, flowers, fruits, young leaves, mature leaves, old leaves, roots and stems. Expression data were obtained from the Tea Plant Information Archive (TPIA). Red and green indicate higher and lower values, respectively, on the expression scale shown.
